# Mechanisms driving open-closed transitions and dimer stability of the large GTPases hGBP1 and hGBP5

**DOI:** 10.3389/fmolb.2026.1732916

**Published:** 2026-05-25

**Authors:** Bastian F. Bundschuh, Jennifer Loschwitz, Birgit Strodel

**Affiliations:** 1 Faculty of Mathematics and Natural Sciences, Heinrich Heine University Düsseldorf, Düsseldorf, Germany; 2 Institute of Biological Information Processing: Structural Biochemistry (IBI-7), Forschungszentrum Jülich, Jülich, Germany

**Keywords:** coiled-coil formation, dynamin-like proteins (DLPs), guanylate-binding proteins (GBPs), large GTPases, molecular dynamics simulations (MD), open-closed transition, protein dimerization

## Abstract

The human guanylate-binding proteins 1 and 5 (hGBP1/5) are key players in innate immunity, vital for defending against intracellular pathogens and mediating membrane-associated immune responses. These protein functions are closely linked to their structural dynamics, making a detailed understanding of the conformational behavior imperative. The closed conformation of hGBP1 is well-characterized through crystallography, but the structural basis for its transition to an active, open state remains less understood. This study uses all-atom and coarse-grained molecular dynamics simulations to investigate the stability and motions of extended monomeric and dimeric hGBP1 and hGBP5 with and without GTP bound. Results reveal that dimers exhibit greater stability than monomers, primarily due to extensive stalk interactions that facilitate a structural crossing of the protomers at the interface of the GTPase and middle domains. This arrangement aligns the middle and effector domains parallel to one another, further stabilizing the dimeric state through the formation of coiled-coil structures supported by salt bridges and hydrophobic contacts. Notably, monomers of both hGBP1 and hGBP5 can revert to a closed state stabilized by a network of salt bridges between the effector domain and the surrounding domains. In hGBP5, this transition is further facilitated by the geranylgeranyl group, which more effectively reaches and buries itself within a hydrophobic pocket of the GTPase domain compared to hGBP1. These findings highlight key factors affecting the stability of hGBP1/5 monomers and dimers, providing insights into their activation mechanisms that are relevant for their role in innate immunity.

## 1 Introduction

Guanylate-binding proteins (GBPs) are a family of dynamin-like large GTPases, expressed in response to interferons, that play essential roles in antimicrobial immunity and can induce cell death—either through pyroptosis or atypical apoptosis—in response to various pathogens (Kutsch and Coers, 2021; Tretina et al., 2019; Fisch et al., 2019). GBPs target a broad range of pathogens, including *Salmonella enterica Typhimurium*, *Shigella flexneri*, *Listeria monocytogenes*, *Mycobacterium bovis*, and *Toxoplasma gondii* (Rivera-Cuevas et al., 2023; Xavier et al., 2020; Kutsch et al., 2020; Rupper and Cardelli, 2008; Wandel et al., 2017; Buijze et al., 2023; Yu et al., 2022; Kravets et al., 2016). Their antimicrobial activity is primarily induced by interferon signaling, leading to upregulation of interferon-stimulated genes, and is characterized by coating microbes during infection (Cheng et al., 1983; Bolen et al., 2014; Kuhm et al., 2024). In humans, particularly hGBP1 and hGBP5 are vital for controling intracellular pathogens. Following recruitment to Gram-negative bacteria, hGBPs inhibit pathogen growth and activate immune responses, including the NLRP3 inflammasome—a protein complex involved in inflammation—and other NOD-like receptors, which are intracellular sensors that detect microbial components and stress signals (Xavier et al., 2020; Johnston et al., 2016).

**FIGURE 1:**
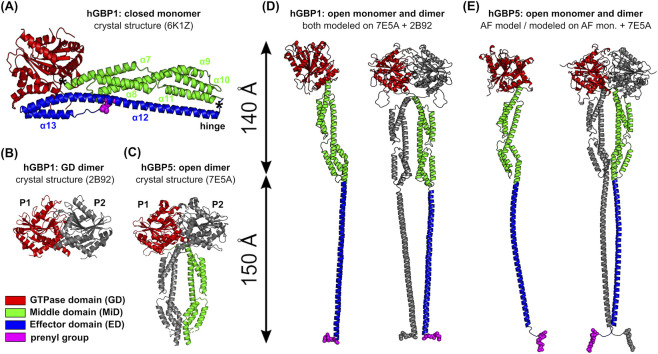
The closed and open structures of hGBP1 and hGBP5 **(A)** The crystal structure of closed hGBP1 (PDB ID: 6K1Z (Vöpel et al., 2010)), **(B)** the crystal structure of the GD dimer of hGBP1 (PDB ID: 2B92 (Ghosh et al., 2006)), **(C)** the crystal structure of the open hGBP5 dimer lacking the ED (PDB ID: 7E5A (Cui et al., 2021)), **(D)** models of the open hGBP1 monomer (left) and dimer (right), **(E)** models of the hGBP5 monomer (left, Alphafold Code: Q96PP8) and dimer (right). In the Methods section, the model-building process for the open forms is described in detail. The GBP domains are colored as follows: red for the GD, green for the MiD, blue for the ED, and magenta for the prenyl groups (farnesyl in hGBP1 and geranylgeranyl in hGBP5). In the dimers, only protomer P1 is colored; protomer P2 is shown in grey for clarity.

The structures of hGBP1 and hGBP5 follow the typical architecture of GBPs, comprising a large GTPase domain (G domain or GD) responsible for GTP binding and hydrolysis, a middle domain (M domain or MiD), and a GTPase effector domain (E domain or ED) for regulation (Figure 1A; Supplementary Figure S1). Both proteins feature a CaaX motif at their C-terminus, which enables isoprenylation—farnesylation in the case of hGBP1 and geranylgeranylation for hGBP5—by which they bind to membranes. This prenylation differentiates them from some other GBPs and dynamin-like proteins in general (Kuhm et al., 2024; Legewie et al., 2019). In the Protein Data Bank (PDB), structures of full-length hGBP1 in its closed form are available for the nucleotide-free state (PDB 1DG3 (Prakash et al., 2000)) and in complex with the GTP analogue GMPPNP (PDB 1F5N (Prakash, 2000)). A similar closed conformation has not yet been observed for hGBP5. In the nucleotide-bound state, both hGBP1 and hGBP5 can adopt an open (or extended) conformation, in which the ED, specifically α12/13 , is detached from the rest of the protein but maintains its helical structure, resulting in an increased overall protein length from approximately 13 to 29 nm (Figure 1) (Peulen et al., 2023; Zhu et al., 2024; Shydlovskyi et al., 2017; Ince et al., 2017; Sistemich et al., 2021). This open state is essential for hGBP1 polymerization across the surface of virulent bacteria, including its binding to the bacterial outer membrane (Zhu et al., 2024; Shydlovskyi et al., 2017), and requires a closed-to-open transition. In the closed, nucleotide-free state, the ED is anchored to the GD via a network of salt bridges involving four glutamate residues (556, 563, 568, 575) within helix α12/13 and residues R227/K228 in helix $\alpha 4^{\prime }$ of the GD (Vöpel et al., 2010). Upon GTP binding, helix $\alpha 4^{\prime }$ shifts position, breaking these salt bridges and promoting ED opening (Vöpel et al., 2010; Weismehl et al., 2024).

Another hGBP1 structure of relevance is that of its GD dimer in complex with GMP/AlF4 (PDB 2B8W (Vöpel et al., 2010), Figure 1B). Using this structure along with that of the closed monomer, we previously built a complete hGBP1 dimer model (Barz et al., 2019). However, hGBP1 coatomers are formed from dimeric units that involve the open protein state (Weismehl et al., 2024; Kuhm et al., 2024). Interestingly, the open hGBP1 dimer involves the same GD–GD interface as the closed dimer (Figure 1D). Additionally, in the open dimer, the two protomers swap their stalk positions within their parallel alignment. This swapping is enabled by crossing the flexible transition regions between the GD and the MiD, which consist of a series of 5–10 residues in coil conformation (Supplementary Figure S2). Transitions between open and closed hGBP1 states are under investigation. Fluorescence resonance energy transfer (FRET) studies suggest that the two GDs first come together, followed by structural opening of the molecules, which then enables interactions between the two MiDs (Sistemich et al., 2021).

For hGBP5, the situation is similar but not identical to that of hGBP1. Both the nucleotide-bound dimer and the monomer (without prenylation) can adopt the open form, as observed by size-exclusion chromatography and coupled with multiangle light scattering (Kutsch et al., 2018). While a dimeric crystal structure for hGBP5 has been reported (PDB 7E5A (Cui et al., 2021)), the authors utilized a C-terminally truncated construct without the ED (residues 1–486) because the full-length protein exhibited significant conformational flexibility and instability. This truncation was necessary to obtain homogeneous species suitable for crystallography, but it leaves the structural arrangement of the two EDs experimentally unresolved in the dimeric state. This structure also reveals swapping of the two MiDs, involving crossing of the coil regions S304–N312 between the GD and MiD domains (Figure 1E; Supplementary Figure S2). Interestingly, for the hGBP5 monomer, Alphafold predicts an open conformation (Figure 1E, Alphafold code Q96PP8), characterized by a long helix comprising α12 and α13 , with no kink or residues adopting a coil structure between them. There are also some differences in the electrostatic potential of hGBP1 and hGBP5, in particular with regard to the ED and the connection between the MiD and the ED (Supplementary Figure S3). This region is composed of ^483^TEKEKEIVE^491^ in hGBP1, while in hGBP5 it involves four consecutive lysine residues with flanking glutamate residues: ^481^TETEKKKKE^489^. Another key difference resides at the C-terminus: hGBP1 harbors the basic ^584^ ${\text{RRRK}}^{587}$ motif, whereas hGBP5 contains the negatively charged ^577^ ${\text{NNDD}}^{580}$ sequence.

The aim of this study is to analyze the stability of the open conformation of hGBP1 and hGBP5 as monomers and dimers. Specifically, we distinguish between the nucleotide-free (apo) state, representing the protein post-hydrolysis, and the GTP-bound (holo) state, which characterizes the ‘primed’ conformation ready for membrane attachment and subsequent polymerization into coatomers. To achieve this, we constructed the relevant monomeric and dimeric models using Alphafold predictions and available experimental structural data (Figures 1D,E), and subjected them to molecular dynamics (MD) simulations using all-atom (AA) and coarse-grained (CG) force fields. To assess protein stability, we quantified their dynamics through various measures and calculated intra- and interprotein interaction energies. The simulation results allow us to delineate the structural characteristics of hGBP1 compared to hGBP5 and propose a sequence for dimer formation and the closed-to-open transition. During this work, open hGBP1 dimer structures became available from cryogenic electron microscopy (cryo-EM) data (Weismehl et al., 2024; Kuhm et al., 2024), which we used to compare with our MD-generated conformations.

## 2 Materials and methods

### 2.1 Model generation

For the generation of the starting structures for the MD simulations, we used the structures as available in the PDB and via Alphafold (AF). Since AF predicts an extended structure for monomeric hGBP5 (AF entry Q96PP8), this was directly employed for the MD simulations and used as template for the extended hGBP1 monomer, which is only available in closed form in the PDB (e.g., PDB entry 6K1Z (Ji et al., 2019)) and also predicted as such by AF. The extension of the ED in the hGBP1 monomer was created in PyMol (Schrödinger, 2025). To generate the extended form of the hGBP5 dimer structure, we used the AF model of the open hGBP5 monomer and combined it with the crystal structure of the hGBP5 dimer lacking the ED (PDB entry 7E5A (Cui et al., 2021)). The latter structure contains a mutation (R356A), which we reverted to the wild-type residue using PyMOL. The two EDs were then added by superimposing two extended hGBP5 monomers onto the mutated hGBP5 dimer crystal structure. The resulting structure served as a template for the hGBP1 dimer in PyMol. Initially, the G domain dimer from PDB structure 2B92 was superimposed onto the hGBP5 dimer, followed by individual superimpositions of the M and E domains. The loops between the GDs and MiDs, specifically where the two protomers cross (S304–N312), had to be manually added. For the holo states, GTP and ${\text{Mg}}^{2+}$ had to be inserted into the active sites of the GDs of hGBP1 and hGBP5, for which we reused the setup from our previous work (Barz et al., 2019). The prenyl groups were added at the EDs using PyMol. The final structures used as starting points for the MD simulations are depicted in Figures 1D,E.

### 2.2 MD simulations

All MD simulations performed in this work were conducted using GROMACS version 2020 (Abraham et al., 2015), employing Amber14SB/TIP3P water (Maier et al., 2015; Jorgensen et al., 1983; Jorgensen, 1981) for all-atom models and MARTINI2/MARTINI water (Marrink et al., 2007) for coarse-grained models. To ensure force-field independence and provide cross-validation of our findings, additional all-atom simulations of the holo-hGBP1/5 monomers and dimers were performed using the CHARMM36 force field in combination with the CHARMM-modified TIP3P water model (Huang and MacKerell, 2013; MacKerell et al., 1998). Different water models were chosen to ensure internal consistency within each respective force field framework. For GTP in AA-MD we used the parameters derived in our previous work (Loschwitz et al., 2023). The parameters for the atomistic presentation of the geranylgeranyl and farnesyl groups were generated using the Antechamber program (Wang et al., 2004; 2006) of the AmberTools15 software package (Case et al., 2005) and ACPYPE (Sousa da Silva and Vranken, 2012) for the simulations together with Amber14SB and CGENFF (Vanommeslaeghe et al., 2009) for the simulations involving CHARMM36. The electron density calculations were performed using Gaussian 09 (Frisch et al., 2009) with the basis set 6-31G* at the Hartree-Fock (HF) level of theory. Partial charges were derived using the RESP (Restrained Electrostatic Potential) method (Bayly et al., 1993; Cornell et al., 1993) following geometry optimization and the electrostatic potential calculations at the HF/6-31G* level.

The CG models of the proteins were created with the Python script *martinize.py* (de Jong et al., 2012) as available at https://cgmartini.nl/docs/downloads/tools/topology-structure-generation.html. This script requires a PDB file of the atomistic structure to be converted along with the secondary structure of this structure, which we determined using the DSSP (Dictionary of Secondary Structure of Proteins) program (Kabsch and Sander, 1983). The CG parameters of the prenyl groups were taken from the work by Atsmon-Raz et al. (Atsmon-Raz and Tieleman, 2017). For the elastic network ElNeDyn applied in MARTINI2, the constraints between the beads were built for distances between 0.5 and 0.9 nm with a force of 500 kJ mol^-1^ nm^-2^ (Periole et al., 2009). Furthermore, we used the domELNEDIN method (Siuda and Thøgersen, 2013) to remove the constraints between the domains, which was applied as Tcl script within the Visual Molecular Dynamics (VMD) program (Humphrey et al., 1996). To maintain neutral conditions Na^+^ ions were added. The system sizes of both monomer and dimer simulations were approximately 1,175,000 atoms for AA-MD and 300,000 beads for CG-MD, comprising proteins, water molecules, and ions.

AA-MD systems underwent energy minimization using the steepest descent method, followed by two equilibration phases in the NVT (100 ps, V-rescale thermostat (Bussi et al., 2007)) and NpT (1 ns, Nosé-Hoover thermostat (Nosé, 1984; Hoover, 1985) and Parinello-Rahman barostat (Parrinello and Rahman, 1982)), which were also applied in the subsequent production runs. The NVT ensemble maintains a constant number of particles (N) , volume (V) , and temperature (T) , while the NpT ensemble keeps N , pressure (p) , and T constant. CG-MD systems were energy-minimized using the steepest descent method. This was followed by two equilibration phases in the NpT ensemble: the first lasted 250 ps and used the Berendsen thermostat (Berendsen et al., 1984) and the second was for 500 ps and employed the V-rescale thermostat (Bussi et al., 2007), with the Parrinello-Rahman barostat applied in both phases. During the CG-MD production runs, the V-rescale thermostat and the Parrinello-Rahman barostat were utilized. During equilibration, all bond lengths were constrained using the LINCS algorithm (Hess et al., 1997), which were released in the production runs apart from the bonds involving hydrogen atoms in AA-MD. The leap-frog algorithm was employed to integrate Newton’s equations of motion, using a timestep of 2 fs for AA simulations and 20 fs for CG simulations. Simulations were conducted at 310 K (37 °C) and 1 bar. To prevent artifacts arising from the rotational diffusion of the ellipsoidal hGBP1/5 monomers and dimers—which could lead to periodic image self-interactions—position restraints were applied to the ${\text{C}}_{\alpha }$ atoms of the β -sheets within the GD during the AA-MD production runs (Barz et al., 2019). The specific residues involved in these restraints are visualized in Supplementary Figure S4, with the corresponding residue numbers provided in the figure caption. In the CG-MD simulations, this was unnecessary, as the smaller number of particles allowed us to create larger, less shape-optimized boxes that avoided such issues.

The production runs conducted in this study are summarized as follows: All AA-MD simulations were 0.5 μ s in duration, comprising single trajectories for apo/holo-hGBP1/5 monomers and dimers using the Amber14SB force field, supplemented by single runs for holo-hGBP1/5 monomers and dimers using CHARMM36. CG-MD simulations of wild-type hGBP1/5 were 5 μ s long and were performed in triplicate for both apo and holo monomers, while single trajectories were collected for the corresponding dimers. Additional individual CG simulations (1 or 5 μ s) were performed to assess the effects of mutations and other variants, as detailed in the Results section. In total, this work is based on an aggregate of 6.5 μ s of AA-MD and 87 μ s of CG-MD sampling.

### 2.3 Analysis

The simulations were analyzed using various tools, including MDAnalysis (Gowers et al., 2016; Michaud-Agrawal et al., 2011), GROMACS tools (Abraham et al., 2015), VMD (Humphrey et al., 1996), as well as custom PyMOL (Schrödinger, 2025) scripts and a plugin tool for the calculation of the electrostatic potential (Jurrus et al., 2017; Dolinsky et al., 2004). Selected snapshots from the CG-MD trajectories were back-mapped to an atomistic representation following the *backward.py* and *initram* workflow (Wassenaar et al., 2014; Wassenaar et al., 2015). Figures were prepared with PyMOL, Inkscape, and BioRender.

The RMSD relative to the initial MD structure was calculated using *gmx rms* for the entire protein and for the individual domains. Before calculating the RMSD of the whole protein, the GD was aligned to its respective starting conformation to focus on the motions of the MiD and ED. For the RMSD calculations of each individual domain, fitting was performed separately for that domain. In the AA-MD simulations, the RMSD was computed for the ${\text{C}}_{\alpha }$ atoms, while in the CG-MD simulations, it was based on the entire backbone.

To monitor the motions of the ED, we measured the distance between the tip of the ED, represented by residue 582, and the last residue of the GD, which is 312. The evolution of this distance was calculated using the GROMACS command *gmx mindist*. The motion of residue 582 as well as residue 480, which is the connection point between the MiD and ED and a potential hinge, was further quantified by analyzing the spatial distribution of these residues during the trajectories using *gmx spatial*. The resulting output was visualized with PyMOL, displaying the spatial distribution of the residues alongside the corresponding MD start and end structures. To analyze the driving forces of the ED motions in the CG-MD simulations, interdomain contacts between the ED and the GD/MiD were calculated using a 6 Å cutoff for the minimum distance between beads of the respective domains. These contacts were time-averaged and are visualized as a heatmap; additionally, specific salt bridge formation rates are explicitly provided.

The binding free energy, ΔG , between the two chains of the dimers of the last 100 ns using 1,000 frames of the AA-MD simulations was calculated using *gmx_MMPBSA* (Valdes-Tresanco et al., 2021). This ΔG was then decomposed into residue–residue interaction energies, $\Delta G_{ij}$ , using first a distance cutoff of 6 Å to identify residues i and j in close proximity. Interaction energies were only further analyzed when $\Delta G_{ij}$ was less than −4 kcal/mol indicating significant attraction between i and j . To characterize the stabilizing interactions within the dimers—particularly the coiled-coil interface between the EDs—residue contacts were identified using a 4 Å distance threshold between the nearest atoms of any two residues. The time-averaged contact probability for each residue pair was calculated, and both intra- and inter-protein contacts were visualized as heatmaps. Furthermore, specific salt-bridge occupancies between the EDs were explicitly extracted. Coiled-coil formation was further quantified by the time evolution of the superhelical radius and the average number of inter-ED contacts during the AA-MD simulations. The superhelical radius represents the average distance of the intertwining helical axes from the superhelix vector. Additionally, the pitch angle and pitch length were calculated for the final frame of each AA-MD trajectory of the dimers (Crick, 1953; Grigoryan and DeGrado, 2011).

## 3 Results

### 3.1 General overview of the protein dynamics

**FIGURE 2:**
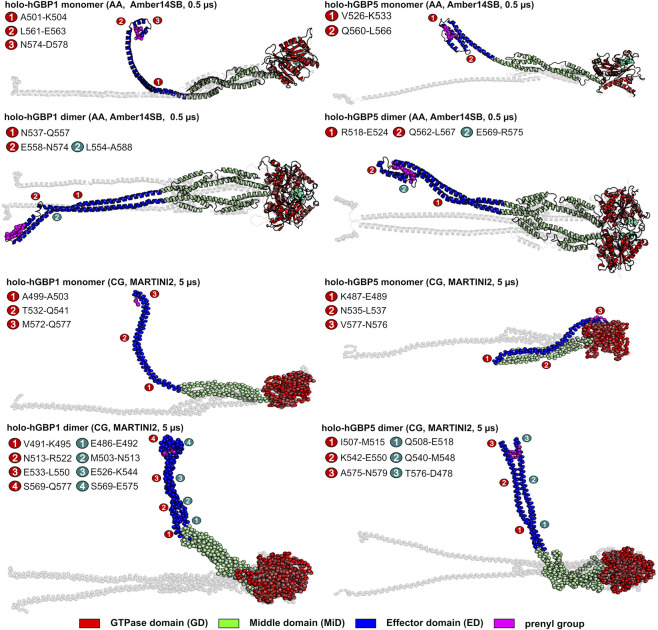
Start and end structures of the MD simulations of holo-hGBP1 (left) and holo-hGBP5 (right) monomers and dimers. Results are shown for the AA-MD (top two plots) and CG-MD (bottom two plots) simulations. The start structures are depicted in gray, while the end structures are shown in color. Regions where helices developed kinks, loops, or turns during the simulations are indicated by circles (red for the first protomer, cyan for the second protomer in the dimers), and the corresponding residue numbers are provided. Similar figures for the simulations of the apo forms, the CHARMM36 simulations, and the replicate CG simulations are available in Supplementary Figures S5-S7.

We begin with a qualitative analysis of the overall stabilities and motions of the extended states of the hGBP1 and hGBP5 monomers and dimers. Although the apo state of the dimer is of lesser biological relevance, it offers valuable insights into whether GTP binding and the prenyl groups directly influence the structural stability of the hGBP1/5 dimers. Figure 2 and Supplementary Figure S5 illustrate the structural changes by presenting the final snapshots from the AA- and CG-MD simulations of the holo and apo proteins, respectively. We identified three main types of motions: (1) a complete open-to-closed transition, bringing the ED to the GD, seen in the monomers of apo-hGBP1/5 and holo-hGBP5 in CG resolution, (2) segmentation of the ED helix at its tip into shorter helices, seen in apo and holo monomers of hGBP1 and hGBP5, and (3) coiled-coil formation between the two EDs in the dimers, seen in the apo and holo dimers of hGBP1 and hGBP5. The motions between the MiD and ED are facilitated by a hinge at the transition from α11 to α12 . This hinge was previously described for hGBP1 (Peulen et al., 2023) and is shown here for the first time explicitly for hGBP5. The flexible nature of the EDs explains why this domain was not visible in a cryo-EM study of hGBP1 (Kuhm et al., 2024) and why an X-ray study of hGBP5 resorted to a truncated protein version lacking the ED (Cui et al., 2021). The segmentation of the ED helix at its C-terminal end occurs—though not exclusively—in regions that lead to the formation of a separated α13 , which is labeled in the closed conformation of hGBP1 in Figure 1A.

We continue with a more detailed explanation of these motions in holo-hGBP1, which are illustrated in the left column of Figure 2 for the Amber14SB (AA-MD) and CG-MD simulations. In the monomer, at AA and CG resolutions, α12 adopts a strongly curved geometry; however, no severe kinking at the hinge between the MiD and ED was observed. Consequently, no open-to-closed transition, as seen for hGBP5, was detected—even after a 5 μ s MD simulation. In the dimer, also no closing occurred, and the ED is less curved. However, considerable overall motions of the protein stalks are still possible, as demonstrated by the CG results. In both AA and CG models, kinks form at the C-terminal ED ends, previously explained by the formation of salt bridges causing the α12 to dissolve at these points (Barz et al., 2019). In the closed hGBP1 conformation we studied earlier, α12 is stabilized through interactions with the GD and MiD (Barz et al., 2019). These supportive tertiary interactions are absent in the open form, leading to structural instability in this helix, which is energetically four times longer than an ideal isolated helix (Qin et al., 2013). As a result, the ED kinks and curves in the open form of the hGBP1 monomer. In the dimer, the two EDs mutually stabilize each other by forming a coiled-coil, thereby confirming previous hypotheses about this interaction (Kutsch et al., 2018). Additionally, the hydrophobic interaction between the two farnesyl groups further enhances the stability of the open dimer form. Our cross-force-field validation demonstrates consistent behavior of the long helical stalks in holo-hGBP1 across both Amber14SB and CHARMM36. While we observed some force-field-dependent dynamics—specifically, the helical breaks at the C-terminus of α 12 are less pronounced in CHARMM36 (Supplementary Figure S6)—the stability of the open state, with the ED undergoing pivoting motions, was consistently captured by both force fields. This robustness is further confirmed by the replicate CG-MD runs (Supplementary Figure S7), underscoring the physical reliability of the observed conformational ensemble.

The structural changes of holo-hGBP5, shown in the right column of Figure 2, reveal that the extended form of hGBP5 is considerably less stable in the monomer than in the dimer. For the monomer, our AA-MD simulations reveal that the ED is characterized by structural instability at its C-terminal end; the helix breaks into shorter segments that pack against one another and interact with the geranylgeranyl group, though they do not fully fold against the MiD or GD within the simulated timeframe. Notably, in the CHARMM36 simulation of the holo-hGBP5 monomer, the open state remained stable throughout the trajectory, with no observed helical breaks (Supplementary Figure S6). At the CG resolution, conversely, the instability of the open state facilitates a complete open-to-closed transition centered at the MiD–ED hinge, an observation consistent across all replicate simulations (Supplementary Figure S7). In one of the three CG-MD runs, this closure involved a 180° turn within the ED centered at residue 528, which promoted contact between the geranylgeranyl group and hydrophobic residues at the MiD C-terminus. The holo-hGBP5 dimer exhibits stability, yet large-scale motions mirroring those of holo-hGBP1. While the dimer maintains an open state, it shows deviations from linearity driven by collective bending of the protein stalks, most notably in the CG-MD simulation due to MiD bending. The C-terminal EDs provide mutual stabilization through a coiled-coil interface, although this interaction is less pronounced than in the holo-hGBP1 dimer.

The dynamics of the apo-hGBP1 and hGBP5 variants underscore the regulatory role of GTP binding and the C-terminal lipid modification. At the all-atom level, both the apo-hGBP1 and hGBP5 monomers exhibit enhanced structural integrity compared to their holo counterparts (Supplementary Figure S5). Specifically, the extended α 12/13 helix exhibits greater stability in its unprenylated state, as the presence of a farnesyl or geranylgeranyl group induces helical instability through direct interactions with the helix. Despite this helical stability at the AA resolution, the CG-MD simulations revealed a full open-to-closed transition for both apo-monomers (Supplementary Figures S5, S7). For apo-hGBP1, this closure was highly robust, occurring in 3/3 replicas—a striking contrast to the holo-hGBP1 monomer, which remained extended in all simulations. For apo-hGBP5, full closure also occurred in all replicates, though similar to the holo state, one of the three apo-hGBP5 CG replicas exhibited a 180° turn within the ED during the closing process, suggesting a conserved transition mechanism. However, the stabilizing interactions following this turn differed by ligand state: in the holo form, the rotation prompted the geranylgeranyl group to interact with hydrophobic residues in the MiD, whereas in the apo form, the negatively charged ^578^ ${\text{DD}}^{579}$ motif interacted with the ^483^ ${\text{KKKK}}^{486}$ cluster at the MiD–ED junction. Notably, this fold-back occurs despite the absence of a canonical turn-inducing sequence (such as Gly or Pro) at the turn point. This suggests that the distal hydrophobic (holo) or electrostatic (apo) ‘clamping’ of the ED termini provide sufficient torque to overcome the local helical propensity of α12 , forcing a localized kinking of the α -helix. In the dimeric state, both apo-hGBP1 and apo-hGBP5 remained in an open conformation across all resolutions. The development of the C-terminal coiled-coil interface in these apo-dimers was closely comparable to the holo-versions.

To further characterize the movement patterns of hGBP1 and hGBP5, we performed an analysis of the spatial distribution function (SDF), which tracks residues by examining the spatial distribution of atoms over the course of the simulation. We monitored the position of the hinge separating the MiD and ED (residue 480) and the tip of the ED (residue 582), resulting in the plots shown in Supplementary Figures S8, and S9 for hGBP1 and hGBP5, respectively. These SDF plots reveal that, at CG resolution, a larger conformational space was sampled—which is expected given the tenfold longer simulation time—and that the protein stalks can move more freely. In contrast, at the AA level, the proteins tend to become trapped in local minima, which are regions of high sampling density. Furthermore, the plots confirm that in the monomers, the EDs explore a larger conformational space than in the dimers. The most significant ED motions occurred in monomeric apo-hGBP1 and apo/holo-hGBP5 during the CG-MD runs, where the protein undergoes open-to-closed transitions that bring the ED into contact with the GD. The SDF plots demonstrate that flexibility is not limited to the MiD–ED hinge; rather, the GD–MiD transition and the MiD itself exhibit significant conformational plasticity. This inherent flexibility leads to significant motions of the MiD, as illustrated by the spatial distribution of residue 480.

### 3.2 Quantification of the protein motions

**FIGURE 3:**
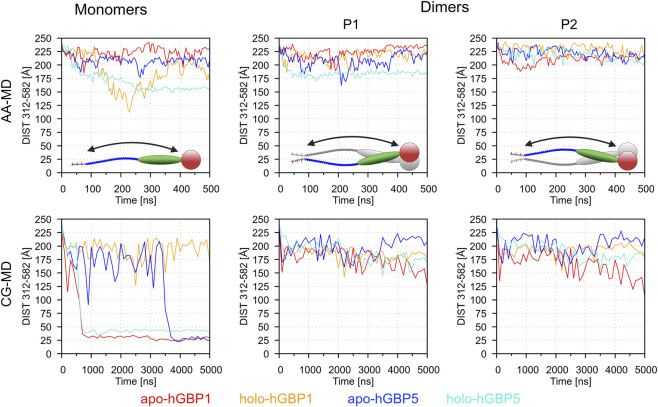
Time evolution of the distance between residue 312 in the GD and residue 582 in the ED in apo-/holo-hGBP1 (red/orange) and apo-/holo-hGBP5 (blue/cyan) during AA-MD (top) and CG-MD (bottom) simulations. The left panels show the results for the monomers, while the middle and right panels depict the results for the two protomers (P1 and P2) of the dimers. The cartoons in the top row panels indicate which distance was measured.

To quantify the motions in hGBP1 and hGBP5 in their different states, we monitored the distance between the tip of the ED and the GD (i.e., between residues 582 and 312). Figure 3 shows the evolution of this distance (called $d_{\mathrm{312-582}}$ in the following) in the apo and holo forms of monomeric and dimeric hGBP1 and hGBP5 during both AA and CG simulations. Starting point was always the open form, corresponding to distances of about 225 Å at time zero, while structural instabilities and the closure of the ED toward the GD resulted in a significant decrease in $d_{\mathrm{312-582}}$ values.

In the AA-MD simulations, where no open-to-closed transitions were observed for any of the monomers, the closest approach between the GD and ED occurred in holo-hGBP1, reaching a minimum distance of $d_{\mathrm{312-582}}\approx 125$ Å. This proximity resulted from collective pivoting of the protein stalk (comprising both the MiD and ED) and was accompanied by localized structural instability within the ED (Supplementary Figure S8). Following complete open-to-closed transitions in the CG-MD simulations of apo-hGBP1 and both apo- and holo-hGBP5, final $d_{\mathrm{312-582}}$ values converged between 25 and 50 Å. Notably, in one of the three replicates for both apo- and holo-hGBP5, the final $d_{\mathrm{312-582}}$ reached approximately 100 Å (Supplementary Figure S10). This distance corresponds to a conformation in which a helical hairpin forms within the ED, positioning the C-terminus near the C-terminal end of the MiD. Interestingly, apo-hGBP5 exhibited slower average closing kinetics (between 0.5 and 4 μ s) than its holo variant (within 1 μ s), suggesting that the interaction between the C-terminal prenyl group and the GD accelerates closure. In contrast, holo-hGBP1 was the only monomer that remained open in CG-MD; although one replicate briefly reached $d_{\mathrm{312-582}}\approx 100$ Å at ≈ 4.4 μ s, it ultimately reverted to an extended state. This suggests that the 15-carbon farnesyl group of hGBP1 is insufficiently long to stabilize a closed conformation, allowing GTP-induced structural changes to maintain the open state. Conversely, the 20-carbon geranylgeranyl group of hGBP5 provides the necessary reach and hydrophobicity to bridge the domain interface and facilitate a stable transition.

In none of the dimers, complete closing is observed. In the 0.5 μ s AA-MD simulations, the dimers stay rather extended, with all distances $d_{\mathrm{312-582}}$ fluctuating between 175 and 230 Å. In the longer 5 μ s CG-MD simulations, more motions are observed, with some $d_{\mathrm{312-582}}$ values falling below 175 Å. The apo-hGBP1 dimer was the least stable in this respect, as at the end of the CG-MD simulations, both chains had reached distances of about 125 Å. The current findings for both hGBP1 monomer and dimer align with previous biochemical results that the open state of hGBP1 should only be acquired following GTP binding (Sistemich et al., 2021).

We further calculated the RMSD from the initial extended states to assess structural changes and determine whether a new stabilized state was reached. For the RMSD of the entire proteins, they were aligned with respect to their GD, whereas for the RMSD of individual domains, they were aligned to their respective starting structures. The time evolution of all RMSD values is shown in Supplementary Figure S11. They indicate that the GD is very stable in all systems with the RMSD fluctuating around 2 Å. For most simulations the RMSD is highest for the ED and can reach values of 15 Å or even higher, while the RMSD of the MiD usually fluctuates between 2 and 5 Å. RMSD deviations are more pronounced in the CG simulations than in the AA-MD, largely because the extended CG timescales allow for broader sampling of the ED conformational space. In systems achieving full closure, the RMSD profiles confirm the acquisition of new, stable conformations. These states—previously identified via visual inspection, SDF, and $d_{\mathrm{312-582}}$ metrics—exhibit greater structural stability than the initial open forms. This is evidenced by the minimal RMSD fluctuations once the closed state is adopted, contrasting with the higher flexibility observed in the various open states of hGBP1 and hGBP5. This suggests that the open state requires additional stabilization, either through the formation of larger protein aggregates or by insertion into a lipid membrane via their prenyl groups.

### 3.3 Interactions during the closing of the monomers

To understand the driving forces behind the closing of the apo-hGBP1 and apo-/holo-hGBP5 monomers sampled during the CG-MD simulations, we analyzed the residue–residue contacts between the ED and the MiD/GD. These interactions are illustrated in Supplementary Figure S12 for the first of the three CG-MD replicates. The observed contacts can be categorized into two groups: the ED interacting with the MiD ( α 6– α 11) and the ED interacting with the GD. Regarding the latter, it is noteworthy that the ED can dock onto the GD at distinct positions. In the CG-MD simulations of apo-hGBP1 and apo-hGBP5, we observed the canonical binding site at helices $\alpha 3/4^{\prime }$ , consistent with the known crystal structure (PDB ID: 6K1Z, Figure 1A). Conversely, the CG-MD run of holo-hGBP5 occupied an alternative site on the opposite side of the GD, involving helices α 0/1. These distinct docking modes—also clearly visible in the SDF plots in Supplementary Figures S8 and S9—were consistently adopted across all CG-MD trajectories where closure occurred (Supplementary Figure S7). The alternative docking site was previously identified via single-molecule FRET spectroscopy studies state of hGBP1 (Peulen et al., 2023). Those studies suggested that this alternative state requires a bending within the MiD, which is consistent with the structural changes observed in our simulations. Furthermore, our results indicate that an ED lacking a kink between α 12 and α 13 is too long to reach the GD in a rigid, linear orientation during closing; consequently, the ED helix adopts a curved conformation to optimize its multivalent interactions with both the MiD and GD.

**FIGURE 4:**
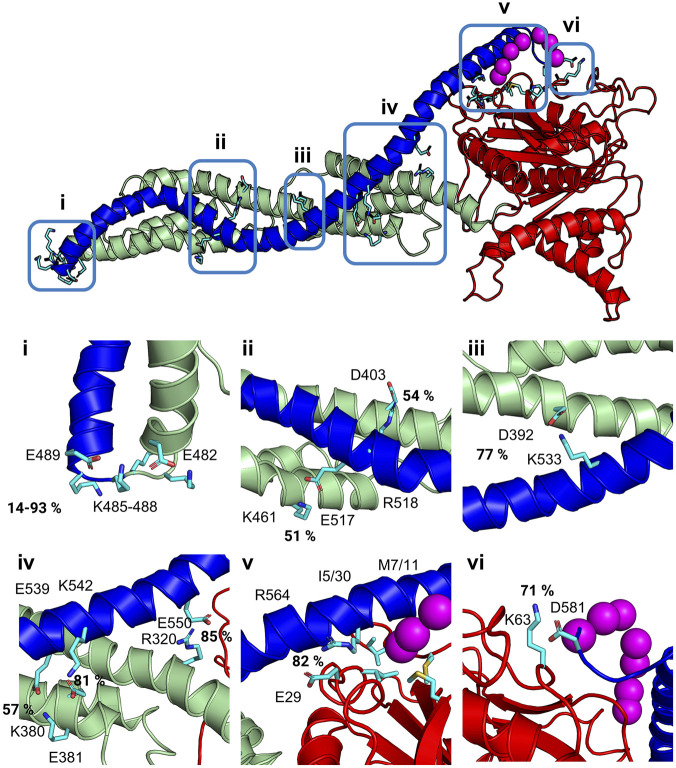
Key interactions driving the open-to-closed transition in the holo-hGBP5 monomer during CG-MD. The protein is shown as a cartoon, with the GD in red, the MiD in green, the ED in blue, and the geranylgeranyl group in magenta. The six regions (i) to (vi), which are of particular relevance, are shown as zoom-ins below the protein. Key residues are depicted as sticks with standard atom-type coloring and labeled by residue number; for each salt bridge, the interaction occupancy is provided as a percentage of the total trajectory duration. The structural representation shows the final frame of a representative CG-MD simulation, back-mapped to the all-atom level from one of three independent replicate trajectories.

The curved ED conformation is clearly visible in the structural representations in Figure 4 and Supplementary Figure S13 for holo-hGBP5 and apo-hGBP1, respectively. These figures also highlight the dominant interactions identified through contact map analysis and salt-bridge occupancies (Supplementary Table S1). In the representative trajectory for holo-hGBP5, the closing process terminates at the alternative α 12/13 binding site on the GD. The primary interaction interfaces between the ED and the MiD/GD are distributed across six distinct regions, labeled (i) through (vi). Closure is initiated by interactions in region (i) at residues 482–489, where the MiD and ED meet. This region undergoes a conformational transition from an extended helix in the open state to a 180° turn in the closed state. The sequence of this region contains a quadruple lysine motif, flanked on either side by glutamate residues—^482^ ${\text{EKKKKE}}^{489}$ —which functions as an electrostatic switch. While its high helical propensity maintains structural integrity in the extended state, the repositioning of these residues during the open-to-closed transition facilitates the formation of new, stabilizing salt bridges across the domain interface. Once the closing motion has commenced, the resulting closed conformation is further stabilized by interactions of the ED—particularly α 12—with both the MiD and GD. The primary stabilizing forces are salt bridges and hydrophobic contacts, including the tethering of the ED helix to the MiD by several consecutive salt bridges: K461–E517, D403–R518, D392–K533, K380–E539, E381–K542, R320–E550, E29–R564, and K63–D581, corresponding to regions (ii) to (vi). Furthermore, the tip of the geranylgeranyl group is stabilized by sequestering within a hydrophobic pocket of the GD (regions (v) and (vi)), composed of isoleucine and methionine residues (I5, I30, M7, and M11) and surrounded by a network of salt bridges. Notably, in the absence of the geranylgeranyl group, the hGBP5 monomer was experimentally found to favor the open conformation (Kutsch et al., 2018). Thus, the presence of the prenyl group and its interaction with the GD hydrophobic pocket serve as significant stabilizing factors for the closed state. Overall, the interactions between the ED and MiD/GD are sufficiently robust that no reverse opening was observed within the current simulation timescales.

Similar observations apply to the closing transition simulated for apo-hGBP1 (Supplementary Figure S13, with regions (i)–(iii) highlighted). Multiple salt bridges form at the hinge region (i) between the MiD and ED. In the central portion of both domains, several additional salt bridges are present, corresponding to region (ii). In region (iii), the ED tethers to the GD via an electrostatic interaction between R584—part of the C-terminal RRR motif—and E164 of Loop 1; this interface is further stabilized by interactions involving polar and hydrophobic residues of the CaaX motif, which is CTIS in hGBP1, and $\alpha 4^{\prime }$ .

To further elucidate the driving forces of the open-to-closed transition, we performed *in silico* mutagenesis studies focusing on the ED termini. For hGBP1, we probed the influence of the C-terminal motif by simulating an apo-hGBP1– Δ CTIS variant (0.5 μ s AA-MD and 5 μ s CG-MD). For holo-hGBP5, we tested the importance of the salt-bridge clusters at the MiD–ED junction by mutating either the flanking residues (K485A/K488A) or the entire polybasic motif (K485A–K488A) (1 μ s CG-MD each). The results in Supplementary Figure S14 indicate that the C-terminus is the primary driver of the closing motion. The apo-hGBP1– Δ CTIS variant remained in an open conformation during CG-MD. In the AA-MD simulation of this variant, α 12 turns at R522–Y524, driven by electrostatic interactions between the C-terminal ^584^ ${\text{RRRK}}^{587}$ motif and the ^458^ ${\text{EE}}^{459}$ motif at the N-terminal end of α 11. While this local rearrangement does not constitute a functional closing transition, as the α 11– α 12 junction remains otherwise intact, the resulting helical hairpin formation in α 12 mirrors the behavior observed in two CG-MD runs of hGBP5 (Supplementary Figure S6). This consistency across different scales and force fields further confirms the reproducibility of our findings.

The lysine mutations in region 485–488 in hGBP5 did not hinder closing; in both the double and quadruple mutants, the open-to-closed transition occurred within 0.5 μ s. We therefore conclude that while the multiple salt bridges between the ED and MiD stabilize the closed conformation, the GTP-binding state and the C-terminal composition are the ultimate determinants of closing. Specifically, GTP binding induces conformational changes in the GD that favor the open state (Vöpel et al., 2010; Sistemich et al., 2021), whereas a longer, more hydrophobic C-terminus (e.g., geranylgeranyl vs. farnesyl in the holo state, or the presence vs. absence of the CaaX motif in the apo state) provides the necessary driving force for closing.

### 3.4 Stabilizing interactions in the dimers

**FIGURE 5:**
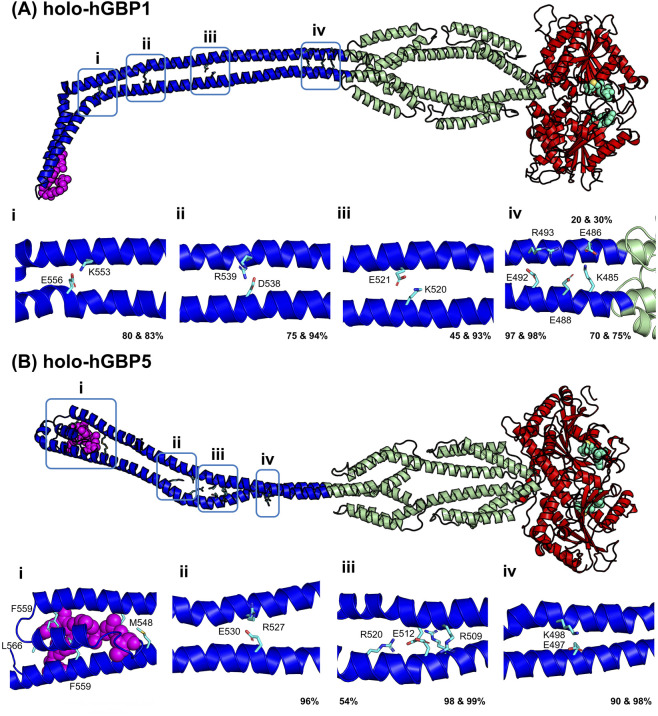
Key interactions between the EDs stabilizing the dimers of **(A)** holo-hGBP1 and **(B)** holo-hGBP5 as determined by MMPBSA applied to the AA-MD simulations. The proteins are shown as a cartoon, with the GD in red, the MiD in green, the ED in blue, and the prenyl groups in magenta. The four regions (i) to (iv), which are of particular relevance per dimer, are shown as zoom-ins below the respective dimer. The side chains of key residues are depicted as sticks with atom-type coloring, and the residue numbers are indicated. For each salt bridge, the time-averaged occupancy is given. When two numbers are given, they correspond to the bidirectional contact frequencies between the symmetric residue pairs across the dimer interface.

Our analyses thus far demonstrate that the extended state in the hGBP1 and hGBP5 monomers is quite flexible, favoring states where the long α12 gets stabilized through contacts with other domains. In the dimers, such stabilization is provided by interprotein interactions, which are examined in more detail in this section. To this end, we applied the Molecular Mechanics Poisson–Boltzmann Surface Area (MM/PBSA) method (Valdes-Tresanco et al., 2021) to the AA-MD simulations of the holo-hGBP1 and holo-hGBP5 dimers. The binding free energies between the two protomers are further decomposed into per-residue contributions $\Delta G_{ij}$ (Supplementary Figure S15A). To identify the key interactions, we applied a $\Delta G_{ij}<-4$ kcal/mol cutoff, yielding four main interaction sites between the two EDs in the dimeric holo-hGBP1 and holo-hGBP5. These sites primarily involve salt bridges and hydrophobic interactions related to the prenyl groups, which are shown in Figure 5. To complement the energetic analysis, we calculated comprehensive contact maps for the dimeric interfaces (Supplementary Figure S16) to determine the occupancy of key salt bridges (Supplementary Table S2). Comparison of the salt-bridge profiles between the apo and holo dimers shows that they are remarkably similar. A key difference is that the cationic motif ^584^ ${\text{RRRK}}^{587}$ in hGBP1 was observed to interact with E568 and E575 in apo-hGBP1. This induces structural breaks between helices α 12 and α 13, which can be seen in Supplementary Figure S5 for the apo-hGBP1 dimer simulated at the all-atom level. Here, breaking point 1 corresponds to the transition between α 12 and α 13. As the remaining contacts between apo and holo dimers are similar, the following analysis focuses on the latter.

The stalks of the holo-hGBP1 dimer are mainly stabilized by the salt bridges located in four regions: (i) K553-E556′, (ii) D538–R539′, (iii) K520–E521′, and (iv) K485–E486′ and E488/E491–R493′, where the prime indicates that the second residue originates from the other protomer (Figure 5A). These salt bridges are 20–30 residues apart from each other and correspond to the intercoil crossings during coiled-coil formation that occurred in the MD simulations of this dimer. Within the holo-hGBP5 dimer shown in Figure 5B, stabilization at the C-terminal end starts in region (i) with hydrophobic interactions between the geranylgeranyl groups with L566, F559, and M548 of both protomers. The extended α12 helices are further stabilized by a salt-bridge between R527 and E530′ in region (ii), followed by a cluster of salt bridges or polar interactions between residues in the range R509–E512 and R520 in region (iii). Because R520 prefers to interact with E512′, this causes a bend in one of the protomers to optimize the arrangement of this salt bridge. The final major stabilizing interaction of the extended structure derives from the salt bridge E497–K498′ in region (iv).

The observations made here align with prediction tools for identifying coiled-coil motifs, such as DeepCoil (Ludwiczak et al., 2019), CoCoPred (Feng et al., 2021), and PCOILS (Gabler et al., 2020), all of which suggest that the two EDs of both hGBP1 and hGBP5 likely adopt a coiled-coil configuration with a probability of up to 95%. In a previous study on hGBP1 investigating the tetramerization of this protein, the coiled-coil formation of the α12 helices was also predicted (Syguda et al., 2012). However, at that time, it was assumed to occur within the closed hGBP1 conformation, with α12 serving as the main interaction site for the self-assembly into a dimer of two hGBP1 dimers. In general, packing interactions in coiled-coil formation typically follow a canonical pattern characterized by a heptad repeat of hydrophobic residues, arranged in parallel or antiparallel orientations, with hydrophobic interactions—specifically, knobs-into-holes packing of the helical stalks—serving as the main driving force, stabilized by salt bridges (Truebestein and Leonard, 2016). Both hGBP1 and hGBP5 loosely contain these motifs (Supplementary Figure S1E), but with a higher number of salt bridges. To further demonstrate that coiled-coil formation indeed occurred, we calculated typical coiled-coil parameters (Crick, 1953; Grigoryan and DeGrado, 2011) including the superhelical radius R_0_, which decreased from 15 Å to below 5 Å, and pitch angles between 5.1° and 11.6° (Supplementary Figure S17; Supplementary Table S2). In stable dimeric coiled-coils, $R_{0}$ typically ranges from 4.9 Å to 5.2 Å and pitch angles from 2° to 15° (Kim et al., 2017). These metrics, alongside a concomitant increase in inter-helical contacts, confirm the transition to a tightly packed dimeric interface across all simulated systems. While the pitch values (111.6–129.4 Å) reside at the lower end of the canonical distribution due to a non-optimized heptad repeat, they clearly indicate the maturation of a characteristic superhelical winding.

Both dimers are further stabilized by interactions between the other two domains, namely, MiD–MiD and GD–GD interactions. This stabilization is illustrated by mapping the $\Delta G_{ij}$ values as color codes onto the protein surface (Supplementary Figure S15B). For the interactions between the two GDs of holo-hGBP1, electrostatic interactions between R244, R245, and D184 as well as hydrophobic contacts involving V71 and V104 are key players, which are partly the same interactions found in the dimer formed by the closed hGBP1 conformation (Schumann et al., 2023). This confirms that the GD–GD interface in the closed and in the extended hGBP1 dimer is very similar. For the MiD–MiD interactions in the extended hGBP1 dimer, stability is provided through polar interactions involving M312, R370, Q419, K423, and S470 and also hydrophobic interactions stemming from L319, V316, and I322. For the holo-hGBP5 dimer, a similar picture emerged: stability of the GD–GD interface is maintained by the positively charged R205, R213, R221, R320, K243, the negatively charged D182, the polar residues Q136, Q242, and the hydrophobic residues Y47, V71, V104. The stabilizing MiD–MiD interactions involve residues, such as N312, R320, K368, K372, K421, S468, as well as hydrophobic residues V314, L317, and F371. The key residues for stabilizing the hGBP1 and hGBP5 dimers in the extended state highlight that salt bridges and hydrophobic interactions are the main driving forces for dimerization, with the GD playing a more significant role than the MiD, alongside the ED–ED interactions discussed above.

**FIGURE 6:**
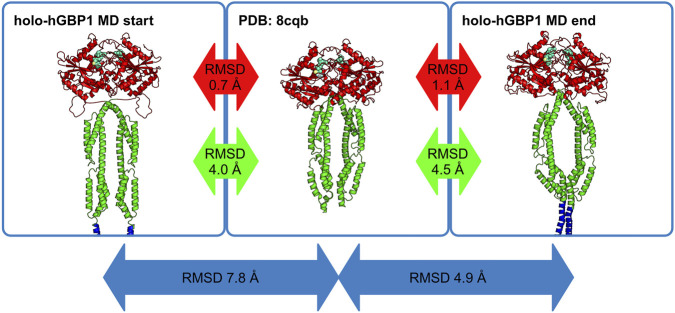
Comparison of hGBP1 dimer structures. The cryo-EM hGBP1 structure 8CQB (Kuhm et al., 2024) (middle), containing the GD and MiD, was aligned to both the model generated for our MD simulations (left) and the resulting AA-MD end structure (right). RMSDs were calculated for the combined GD and MiD (values at the bottom), as well as for each domain individually after their separate alignment: GD in red and MiD in green.

During this work, a cryo-EM structure of the holo-hGBP1 dimer became available, yet containing only the GD and MiD (PDB 8CQB (Kuhm et al., 2024)). In Figure 6, this structure is compared to our initial hGBP1 dimer model and to the dimer structure at the end of the 0.5 μ s AA-MD simulation of the holo-hGBP1 dimer. The RMSD between the GD plus MiD of the cryo-EM structure and our initial model is 7.8 Å, while it is reduced to 4.9 Å when compared to the final MD state. The reduction in RMSD during the MD simulation indicates that the AA-MD successfully refined our initial hGBP1 dimer model toward a structure more consistent with cryo-EM data. We also calculated the RMSDs between the individual domains after separate alignment to only the GD or MiD, which resulted in lower values. The RMSD of the GD, comparing the MD start and end structures to the cryo-EM structure, is 0.7 Å and 1.1 Å, respectively. This demonstrates that the GD–GD interface is stable, as a larger increase in RMSD would otherwise have been observed. Here, it should be noted that in AA-MD positional restraints were applied to the β -sheets of the GDs (Supplementary Figure S4) to prevent periodic image self-interactions arising from rotational protein diffusion. While these restraints inherently prevent dimer dissociation, their influence on the conformational dynamics is negligible. This is supported by previous in-depth analyses of GD dynamics under identical restraint conditions (Barz et al., 2019), as well as our current unrestrained CG-MD simulations, in which the GD–GD interface remained intrinsically stable over significantly longer timescales.

For the MiD, the corresponding RMSD values are 4.0 Å and 4.5 Å, respectively. These deviations from the cryo-EM structure originate from opposing arrangements of the two MiDs in the dimer: while in our initial model the two α9 are quite close to each other, they moved apart during the simulation, and α8 and α10 became bent. In particular, the two α11 moved toward each other and now form an interface, which facilitates coiled-coil formation in the subsequent α12 of the ED. Overall, the arrangement of the helices in the MiD of the MD end structure is more similar to the cryo-EM structure than in the MD start structure. Only the distance between the two α9 became too large, explaining the larger RMSD for the MiD in the MD end structure compared to the start. Nevertheless, the overall RMSD is lower (4.9 Å vs. 7.8 Å), confirming that the arrangement of the structural units improved during MD.

The recently released PDB entry 8R1A provides a structural model of the full hGBP1 dimer within a membrane-bound oligomer (Weismehl et al., 2024). However, it is important to distinguish this template from an experimentally determined assembly; entry 8R1A represents a model generated via SWISS-Model using a truncated hGBP5 dimer crystal structure (PDB 7E5A (Cui et al., 2021)) and Alphafold predictions. While we employed a similar approach to generate our starting configuration (Figure 1), our multi-scale MD approach moves beyond static modeling to interrogate the physical interactions and conformational transitions of the distal domains. Notably, our simulations reveal that the EDs do not merely remain in proximity—as seen in the non-interacting helices of the 8R1A model—but instead undergo a spontaneous transition to form a stable coiled-coil interface. By providing a physically grounded trajectory for this interaction, our work offers a mechanistic explanation for the stabilization of the open dimer, transforming a hypothesized orientation into a dynamically validated structural scaffold.

Another region that warrants closer inspection is the loop connecting the GD to the MiD, which is crucial because it involves the crossing of the two protomers; specifically, the MiD and ED of the protomer shown on the left in Figure 6 belong to the GD on the right side. This loop region had to be manually modeled in our starting structure (Supplementary Figure S2) and initially deviates significantly from the cryo-EM structure. However, during MD, this loop adopts a more realistic conformation. In particular, an important electrostatic contact was established between K308 in the coil region between the GD and MiD and K155 in the GD, further stabilized by the hydrophobic interaction between L309 and W114. It has been shown that mutations in the coil region between the GD and MiD—namely D308S, L309A, and P310A—disrupt this interface, inhibiting the formation of the extended dimer and thus preventing membrane association (Kuhm et al., 2024). The swapping of the two protein stalks is further stabilized through interactions between residues of one protomer’s MiD and residues of the other protomer’s GD, such as R151–D323′ and N161–R370′ in hGBP1, and E147/D150–K372′, R155–D321′, and R162–E364′ in hGBP5.

## 4 Discussion

We investigated the structural and dynamic differences between extended monomeric and dimeric hGBP models, focusing on stability, movement patterns, and key residues involved in monomer closure and dimer stabilization. To achieve this, we performed all-atom MD (AA-MD) simulations using the Amber14SB and CHARMM36 force fields, as well as coarse-grained MD (CG-MD) using the MARTINI2.2 force field, focusing on both the apo and holo states of hGBP1 and hGBP5. The use of high- and low-resolution force fields provides a comprehensive perspective on the behavior of these long-helical proteins. While the initial models incorporated Alphafold-predicted structures, the subsequent MD simulations provided a necessary refinement step. The high degree of agreement across force fields and convergence with independent experimental data for the hGBP1 dimer (PDB 8CQB) indicate that the conformational ensembles reported here are robust and represent physically grounded states rather than template-driven artifacts. To address sampling limitations and potential kinetic trapping inherent in all-atom simulations of large systems, we utilized a multi-scale approach. Global conformational space was explored via CG-MD, while the local thermodynamic stability and fine-grained interaction networks were validated through AA-MD trajectories. The convergence of structural observables across replicate CG-MD simulations confirms the robustness of the reported conformational states.

### 4.1 Structural determinants of open hGBP stability and transitions

**FIGURE 7:**
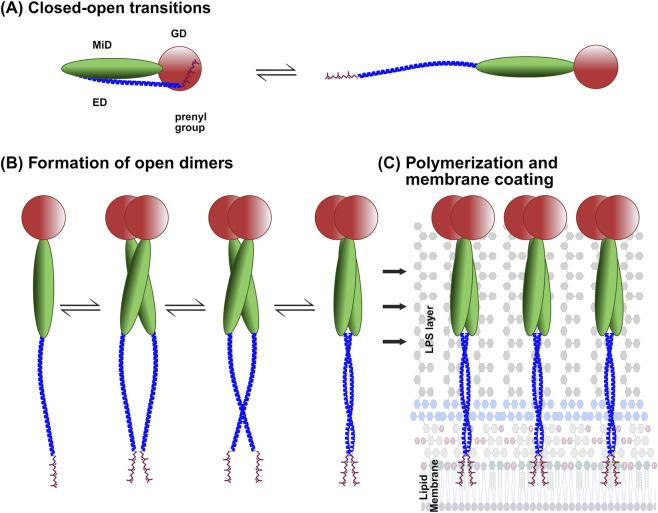
Schematic overview of current results and perspectives on biological relevance. **(A)** Monomeric hGBP1/5 can switch between the closed and open states. **(B)** The extended hGBP1/5 dimers are stabilized through MiD/ED domain swapping and coiled-coil formation between the two ED helices. **(C)** The extended hGBP1/5 dimers remain flexible and are stabilized further through interactions with other hGBP1/5 dimers. In the case of hGBP1 it was shown that this leads to membrane coating and interactions with the O-antigen chains of LPS molecules (Zhu et al., 2024).

Our results demonstrate that monomeric extended hGBP1 and hGBP5 structures are inherently less stable than their dimeric counterparts (Figure 7A). Dimers are stabilized by extensive interactions within their stalks and a characteristic crossing at the GD–MiD transition point (Figure 7B). This crossing facilitates stabilizing contacts between protomers when aligned parallel to each other. As revealed by our CG-MD simulation of an Alphafold-predicted mGBP2 dimer—which lacks this domain swapping—parallel alignment alone is insufficient for stability (Supplementary Figure S18). Further stabilization is achieved through coiled-coil formation of the two EDs; this arrangement buries hydrophobic residues while exposing a polar surface to the solvent, optimizing the stalks for interaction with the O-antigen chains of bacterial lipopolysaccharides (LPS).

Without these inter-protein interactions, the stalks in the monomers display significant hinge motions that can lead to a complete open-to-closed transition. However, compared to previous work analyzing dimeric GBPs in closed conformations (Schumann et al., 2023), the open dimer structures also exhibit significantly greater flexibility. Since the GD–GD interaction interface is largely identical in both closed and open dimers (PDB 2B92 (Ghosh et al., 2006) vs. 8CQB (Kuhm et al., 2024)), these dynamic differences are not attributable to the head-group interface but rather derive from the inherent conformational plasticity of the long protein stalks in the open state.

This study’s CG-MD simulations provide mechanistic insight into the open-to-closed transition of hGBP1/5 monomers. We observed complete transitions in monomeric apo-hGBP1, as well as both apo- and holo-hGBP5. The process is initiated by a helix-to-turn conversion at the MiD–ED junction. In both proteins, this region contains several charged residues—specifically a quadruple lysine with flanking glutamate motif in hGBP5—that functions as an electrostatic switch. While their high intrinsic helical propensity maintains structural integrity in the extended state, the repositioning of these residues during the transition facilitates the formation of new salt bridges across the domain interface. In some of the replicate simulations, the ED underwent a helix-to-helical hairpin transition, involving a 180° turn within the ED itself. This conformation is stabilized by favorable interactions between the C-terminus and the MiD.

We found that prenyl group length significantly influences closure kinetics. Apo-hGBP5 exhibited slower average closing compared to its holo variant, suggesting that C-terminal prenyl interactions with the GD accelerate the process. The 20-carbon geranylgeranyl group provides the necessary reach to bridge the domain interface and sequester within a hydrophobic GD pocket. In contrast, holo-hGBP1 remained open in CG-MD; although it showed transient tendencies to close, it consistently reverted to an extended state. This suggests that the 15-carbon farnesyl group of hGBP1 is insufficiently long to establish the persistent interactions required to stabilize a closed state, allowing GTP-induced structural changes to favor the open conformation. Further supporting the role of the C-terminus in structural regulation, our simulations demonstrated that shortening the protein by removing the final four residues—thereby reducing C-terminal hydrophobicity—effectively prevents closure in apo-hGBP1. We propose that this finding represents a testable hypothesis for wet-lab validation, where the use of truncated hGBP1 variants could confirm whether C-terminal length and hydrophobicity are the primary gatekeepers of the open-to-closed transition.

The closing process terminates with the ED binding either at the canonical $\alpha 3/4^{\prime }$ site, consistent with the known crystal structure of closed hGBP1 (PDB ID: 6K1Z (Vöpel et al., 2010)), or at an alternative α 0/1 binding site, consistent with observations from a FRET spectroscopy study (Peulen et al., 2023). Conversely, the reverse closed-to-open transition likely requires GTP binding (Vöpel et al., 2010; Sistemich et al., 2021; Weismehl et al., 2024), which induces conformational shifts in the GD that translate into structural changes along the protein stalk. Our previous studies showed that GTP binding reduces guanine-binding cap motion and lowers the entropic barrier for dimerization (Barz et al., 2019; Schumann and Strodel, 2023), which would facilitate protein opening as the protomers stabilize each other. Our morphing model (Supplementary Movie S1) suggests a concerted transition where stalk crossing and unfolding occur simultaneously. Given that hGBP1/5 hydrolyze GTP to GMP, the initial hydrolysis step to GDP may provide the necessary energy for these large-scale shifts (Schwemmle and Staeheli, 1994; Kunzelmann et al., 2006; Kutsch et al., 2018). Further research is essential to clarify the energy requirements for unfolding the monomers and dimers and to determine how GTP hydrolysis, a process that was not studied here, influences these conformational states.

### 4.2 Structural basis for the functional divergence of hGBP1 and hGBP5

The conformational transitions observed in our MD simulations provide a structural framework for understanding the diverse functional roles of hGBP1 and hGBP5 in innate immunity. The closed-to-open transition represents a critical regulatory mechanism that governs membrane targeting through controlled exposure of the C-terminal CaaX motif. In the closed conformation, the farnesyl group remains sequestered within the protein interior; upon opening, we propose that this prenylated anchor becomes solvent-accessible, potentially enabling high-affinity insertion into hydrophobic membrane environments such as bacterial LPS membranes or viral replication vesicles.

Our simulations revealed that domain swapping and coiled-coil formation in the E domains are the key structural events stabilizing the open conformation. For hGBP1, this transition appears to serve dual anchoring functions: first, prenyl exposure facilitates insertion into the hydrophobic core of LPS membranes; second, the conformational rearrangement aligns the polybasic motif ^582^ ${\text{KMRRRK}}^{587}$ at the C-terminal tip for electrostatic engagement with anionic membrane phosphates (Figure 7C). Critically, upon dimerization in the open state, this local charge density doubles to ten basic residues, creating a multivalent electrostatic anchor with high avidity for the polyanionic phosphate groups of bacterial Lipid A. This computational prediction provides a structural bridge for established biochemical observations: hGBP1 binds LPS directly and independently of other cytosolic factors (Santos et al., 2020), which is driven by electrostatic forces (Dickinson et al., 2023), and the RRR motif at positions 584–586 is essential for LPS membrane association (Kutsch et al., 2020; Zhu et al., 2024). The hGBP1-R584–586A mutant fails to localize to LPS-containing membranes and exhibits impaired recruitment of Caspase-4/11 (Kutsch et al., 2020; Zhu et al., 2024). Importantly, truncations of the LPS O-antigen polysaccharide do not block hGBP1 membrane association or high-order coat formation (Kutsch et al., 2020; Zhu et al., 2024), showing that the polybasic motif—rather than specific saccharide recognition—is the primary determinant of membrane targeting.

The stabilized open dimer conformation observed in our MD trajectories thus likely represents the molecular trigger that clusters these ten basic residues at the C-terminal tip of hGBP1, positioning them for multivalent engagement with bacterial membranes. This structural arrangement is potentially essential for the formation of supramolecular hGBP1 coats on intracellular pathogens, a critical antimicrobial mechanism. Dimerization is known to be required for coat assembly on LPS membranes and subsequent bacterial lysis (Zhu et al., 2024); our simulations contextualize these findings by revealing the atomic-level structural basis for dimer stability and the conformational prerequisites for coat nucleation.

As a monomer, hGBP5 exhibits reduced stability in the open conformation compared to hGBP1. Sequence alignment reveals a striking electrostatic reversal at the C-terminal tip: where hGBP1 possesses the basic ^586^ ${\text{RK}}^{587}$ motif, hGBP5 contains two negatively charged aspartate residues (^580^ ${\text{DD}}^{581}$ ). This substitution likely renders direct, high-affinity binding to the polyanionic Lipid A moiety of LPS energetically unfavorable for hGBP5. These findings support a model in which hGBP5 functions primarily as a signaling scaffold rather than a membrane-disrupting coating protein, a distinction consistent with its established role as an NLRP3 inflammasome regulator (Shenoy et al., 2012; Kim et al., 2016).

We hypothesize that for hGBP5, the achievement of specific, transient domain orientations—rather than the prolonged stability of an open state—is the primary requirement for productive interactions with inflammasome machinery or Golgi-resident viral proteins. Consequently, the differential conformational dynamics observed in our simulations likely reflect the functional specialization of these isoforms: hGBP1 is optimized for a stable, electrostatically potent open dimer necessary for pathogen coating, whereas hGBP5 utilizes a more fluid conformational landscape to orchestrate protein–protein interactions within inflammatory signaling cascades. These results underscore how subtle variations in primary sequence and conformational equilibrium encode the remarkable functional diversity of the GBP family in innate immune defense.

## 5 Conclusion

This study investigated the stability and dynamics of extended monomeric and dimeric hGBP1 and hGBP5 structures using all-atom and coarse-grained MD simulations. The dimers were found to be significantly more stable than the monomers, primarily due to extensive interactions between the long stalks of the two protomers. Open monomers exhibited substantial flexibility, with open-to-closed transitions occurring in all variants except for holo-hGBP1 and a C-terminally truncated apo-hGBP1 mutant. These collective findings for monomeric hGBP1 and hGBP5 indicate that both the length of the E domain and the hydrophobicity of its C-terminus act as critical determinants in the kinetics of the open-to-closed transition. Stability in the dimeric forms is achieved through a structural crossing of the two protomers at the G–M domain interface, which optimizes parallel alignment and facilitates stabilizing interactions between the M and E domains. The E domains further enhance dimer integrity by forming a coiled-coil structure, enabling key salt bridges and hydrophobic contacts between the stalks. These results provide a structural rationale for why hGBP1 may require additional polymerization and engagement with lipid membranes to fully stabilize its open conformation for pathogen defense. Our future research will focus on elucidating these higher-order stabilization interactions and providing further molecular insights into hGBP recruitment to pathogenic membranes.

## Data Availability

The raw data supporting the conclusions of this article will be made available by the authors, without undue reservation.

## References

[B1] Abraham M. J. Murtola T. Schulz R. Páll S. Smith J. C. Hess B. (2015). GROMACS: high performance molecular simulations through multi-level parallelism from laptops to supercomputers. SoftwareX 1-2, 19–25. 10.1016/j.softx.2015.06.001

[B2] Atsmon-Raz Y. Tieleman D. P. (2017). Parameterization of palmitoylated cysteine, farnesylated cysteine, geranylgeranylated cysteine, and myristoylated glycine for the martini force field. J. Phys. Chem. B 121, 11132–11143. 10.1021/acs.jpcb.7b10175 29144135

[B3] Barz B. Loschwitz J. Strodel B. (2019). Large-scale, dynamin-like motions of the human guanylate binding protein 1 revealed by multi-resolution simulations. PLOS Comput. Biol. 15, e1007193. 10.1371/journal.pcbi.1007193 31589600 PMC6797221

[B4] Bayly C. I. Cieplak P. Cornell W. Kollman P. A. (1993). A well-behaved electrostatic potential based method using charge restraints for deriving atomic charges: the resp model. J. Phys. Chem. 97, 10269–10280. 10.1021/j100142a004

[B5] Berendsen H. J. C. Postma J. P. M. van Gunsteren W. F. DiNola A. Haak J. R. (1984). Molecular dynamics with coupling to an external Bath. J. Chem. Phys. 81, 3684–3690. 10.1063/1.448118

[B6] Bolen C. R. Ding S. Robek M. D. Kleinstein S. H. (2014). Dynamic expression profiling of type i and type iii interferon-stimulated hepatocytes reveals a stable hierarchy of gene expression: hepatology. Hepatology 59, 1262–1272. 10.1002/hep.26657 23929627 PMC3938553

[B7] Buijze H. Brinkmann V. Hurwitz R. Dorhoi A. Kaufmann S. H. E. Pei G. (2023). Human gbp1 is involved in the repair of damaged phagosomes/endolysosomes. Int. J. Mol. Sci. 24, 9701. 10.3390/ijms24119701 37298652 PMC10253393

[B8] Bussi G. Donadio D. Parrinello M. (2007). Canonical sampling through velocity rescaling. J. Chem. Phys. 126, 014101. 10.1063/1.2408420 17212484

[B9] Case D. A. Cheatham T. E. Darden T. Gohlke H. Luo R. Merz K. M. (2005). The amber biomolecular simulation programs. J. Comput. Chem. 26, 1668–1688. 10.1002/jcc.20290 16200636 PMC1989667

[B10] Cheng Y. S. Colonno R. J. Yin F. H. (1983). Interferon induction of fibroblast proteins with guanylate binding activity. J. Biol. Chem. 258, 7746–7750. 10.1016/s0021-9258(18)32242-7 6305951

[B11] Cornell W. D. Cieplak P. Bayly C. I. Kollman P. A. (1993). Application of resp charges to calculate conformational energies, hydrogen bond energies, and free energies of solvation. J. Am. Chem. Soc. 115, 9620–9631. 10.1021/ja00074a030

[B12] Crick F. H. (1953). The packing of *α*-helices: simple coiled-coils. Acta Crystallographica 6, 689–697. 10.1107/s0365110x53001964

[B13] Cui W. Braun E. Wang W. Tang J. Zheng Y. Slater B. (2021). Structural basis for GTP-Induced dimerization and antiviral function of guanylate-binding proteins. Proc. Natl. Acad. Sci. U. S. A. 118, e2022269118. 10.1073/pnas.2022269118 33876762 PMC8054025

[B14] de Jong D. H. Singh G. Bennett W. F. D. Arnarez C. Wassenaar T. A. Schäfer L. V. (2012). Improved parameters for the martini coarse-grained protein force field. J. Chem. Theory Comput. 9, 687–697. 10.1021/ct300646g 26589065

[B15] Dickinson M. S. Kutsch M. Sistemich L. Hernandez D. Piro A. S. Needham D. (2023). Lps-aggregating proteins gbp1 and gbp2 are each sufficient to enhance caspase-4 activation both in cellulo and *in vitro* . Proc. Natl. Acad. Sci. 120, e2216028120. 10.1073/pnas.2216028120 37023136 PMC10104521

[B16] Dolinsky T. J. Nielsen J. E. McCammon J. A. Baker N. A. (2004). Pdb2pqr: an automated pipeline for the setup of poisson-boltzmann electrostatics calculations. Nucleic Acids Res. 32, W665–W667. 10.1093/nar/gkh381 15215472 PMC441519

[B17] Feng S.-H. Xia C.-Q. Shen H.-B. (2021). Cocopred: coiled-coil protein structural feature prediction from amino acid sequence using deep neural networks. Bioinformatics 38, 720–729. 10.1093/bioinformatics/btab744 34718416

[B18] Fisch D. Bando H. Clough B. Hornung V. Yamamoto M. Shenoy A. R. (2019). Human gbp1 is a microbe-specific gatekeeper of macrophage apoptosis and pyroptosis. EMBO J. 38, e100926. 10.15252/embj.2018100926 31268602 PMC6600649

[B19] Frisch M. J. Trucks G. W. Schlegel H. B. Scuseria G. E. Robb M. A. Cheeseman J. R. (2009). Gaussian 09 revision E.01.

[B20] Gabler F. Nam S. Till S. Mirdita M. Steinegger M. Söding J. (2020). Protein sequence analysis using the mpi bioinformatics toolkit. Curr. Protoc. Immunol. 72, e108. 10.1002/cpbi.108 33315308

[B21] Ghosh A. Praefcke G. J. K. Renault L. Wittinghofer A. Herrmann C. (2006). How guanylate-binding proteins achieve assembly-stimulated processive cleavage of gtp to gmp. Nature 440, 101–104. 10.1038/nature04510 16511497

[B22] Gowers R. Linke M. Barnoud J. Reddy T. Melo M. Seyler S. (2016). “Mdanalysis: a python package for the rapid analysis of molecular dynamics simulations,” in Proceedings of the 15th python in science conference (SciPy), 98–105. 10.25080/majora-629e541a-00e

[B23] Grigoryan G. DeGrado W. F. (2011). Probing designability *via* a generalized model of helical bundle geometry. J. Mol. Biol. 405, 1079–1100. 10.1016/j.jmb.2010.08.058 20932976 PMC3052747

[B24] Hess B. Bekker H. Berendsen H. J. C. Fraaije J. G. E. M. (1997). Lincs: a linear constraint solver for molecular simulations. J. Comput. Chem. 18, 1463–1472. 10.1002/(sici)1096-987x(199709)18:12⟨1463::aid-jcc4⟩3.0.co;2-h

[B25] Hoover W. G. (1985). Canonical dynamics: equilibrium phase-space distributions. Phys. Rev. A 31, 1695–1697. 10.1103/PhysRevA.31.1695 9895674

[B26] Huang J. MacKerell A. D. (2013). Charmm36 all-atom additive protein force field: validation based on comparison to nmr data. J. Comput. Chem. 34, 2135–2145. 10.1002/jcc.23354 23832629 PMC3800559

[B27] Humphrey W. Dalke A. Schulten K. (1996). VMD: visual molecular dynamics. J. Mol. Graph. 14, 33–38. 10.1016/0263-7855(96)00018-5 8744570

[B28] Ince S. Kutsch M. Shydlovskyi S. Herrmann C. (2017). The human guanylate-binding proteins hgbp-1 and hgbp-5 cycle between monomers and dimers only. FEBS J. 284, 2284–2301. 10.1111/febs.14126 28580591

[B29] Ji C. Du S. Li P. Zhu Q. Yang X. Long C. (2019). Structural mechanism for guanylate-binding proteins (gbps) targeting by the shigella e3 ligase ipah9.8. PLOS Path 15, e1007876. 10.1371/journal.ppat.1007876 31216343 PMC6602295

[B30] Johnston A. C. Piro A. Clough B. Siew M. Virreira Winter S. Coers J. (2016). Human gbp1 does not localize to pathogen vacuoles but restrictstoxoplasma gondii. Cell. Microbiol. 18, 1056–1064. 10.1111/cmi.12579 26874079 PMC4961618

[B31] Jorgensen W. L. (1981). Quantum and statistical mechanical studies of liquids. 10. Transferable intermolecular potential functions for water, alcohols, and ethers. Application to liquid water. J. Am. Chem. Soc. 103, 335–340. 10.1021/ja00392a016

[B32] Jorgensen W. L. Chandrasekhar J. Madura J. D. Impey R. W. Klein M. L. (1983). Comparison of simple potential functions for simulating liquid water. J. Chem. Phys. 79, 926–935. 10.1063/1.445869

[B33] Jurrus E. Engel D. Star K. Monson K. Brandi J. Felberg L. E. (2017). Improvements to the apbs biomolecular solvation software suite. Protein Sci. 27, 112–128. 10.1002/pro.3280 28836357 PMC5734301

[B34] Kabsch W. Sander C. (1983). Dictionary of protein secondary structure: pattern recognition of hydrogen-bonded and geometrical features. Biopolymers 22, 2577–2637. 10.1002/bip.360221211 6667333

[B35] Kim B.-H. Chee J. D. Bradfield C. J. Park E.-S. Kumar P. MacMicking J. D. (2016). Interferon-induced guanylate-binding proteins in inflammasome activation and host defense. Nat. Immunol. 17, 481–489. 10.1038/ni.3440 27092805 PMC4961213

[B36] Kim B.-W. Jung Y. O. Kim M. K. Kwon D. H. Park S. H. Kim J. H. (2017). Accord: an assessment tool to determine the orientation of homodimeric coiled-coils. Sci. Rep. 7, 43318. 10.1038/srep43318 28266564 PMC5339707

[B37] Kravets E. Degrandi D. Ma Q. Peulen T.-O. Klümpers V. Felekyan S. (2016). Guanylate binding proteins directly attack Toxoplasma gondii *via* supramolecular complexes. eLife 5, e11479. 10.7554/elife.11479 26814575 PMC4786432

[B38] Kuhm T. Taisne C. de Agrela Pinto C. Gross L. Giannopoulou E. A. Huber S. T. (2024). Structural basis of antimicrobial membrane coat assembly by human gbp1. Nat. Struct. Mol. Biol. 32, 172–184. 10.1038/s41594-024-01400-9 39394410 PMC11746146

[B39] Kunzelmann S. Praefcke G. J. Herrmann C. (2006). Transient kinetic investigation of gtp hydrolysis catalyzed by interferon-*γ*-induced hgbp1 (human guanylate binding protein 1). J. Biol. Chem. 281, 28627–28635. 10.1074/jbc.M604911200 16873363

[B40] Kutsch M. Coers J. (2021). Human guanylate binding proteins: nanomachines orchestrating host defense. FEBS J. 288, 5826–5849. 10.1111/febs.15662 33314740 PMC8196077

[B41] Kutsch M. Ince S. Herrmann C. (2018). Homo and hetero dimerisation of the human guanylate-binding proteins hgbp-1 and hgbp-5 characterised by affinities and kinetics. FEBS J. 285, 2019–2036. 10.1111/febs.14459 29618166

[B42] Kutsch M. Sistemich L. Lesser C. F. Goldberg M. B. Herrmann C. Coers J. (2020). Direct binding of polymeric GBP1 to LPS disrupts bacterial cell envelope functions. EMBO J. 39, e104926. 10.15252/embj.2020104926 32510692 PMC7327485

[B43] Legewie L. Loschwitz J. Steffens N. Prescher M. Wang X. Smits S. H. J. (2019). Biochemical and structural characterization of murine GBP7, a guanylate binding protein with an elongated c-terminal tail. Biochem. J. 476, 3161–3182. 10.1042/bcj20190364 31689351

[B44] Loschwitz J. Steffens N. Wang X. Schäffler M. Pfeffer K. Degrandi D. (2023). Domain motions, dimerization, and membrane interactions of the murine guanylate binding protein 2. Sci. Rep. 13, 679. 10.1038/s41598-023-27520-8 36639389 PMC9839784

[B45] Ludwiczak J. Winski A. Szczepaniak K. Alva V. Dunin-Horkawicz S. (2019). Deepcoil—A fast and accurate prediction of coiled-coil domains in protein sequences. Bioinformatics 35, 2790–2795. 10.1093/bioinformatics/bty1062 30601942

[B46] MacKerell A. D. Bashford D. Bellott M. Dunbrack R. L. Evanseck J. D. Field M. J. (1998). All-atom empirical potential for molecular modeling and dynamics studies of proteins. J. Phys. Chem. B 102, 3586–3616. 10.1021/jp973084f 24889800

[B47] Maier J. A. Martinez C. Kasavajhala K. Wickstrom L. Hauser K. E. Simmerling C. (2015). Ff14SB: improving the accuracy of protein side chain and backbone parameters from ff99SB. J. Chem. Theory Comput. 11, 3696–3713. 10.1021/acs.jctc.5b00255 26574453 PMC4821407

[B48] Marrink S. J. Risselada H. J. Yefimov S. Tieleman D. P. de Vries A. H. (2007). The MARTINI force field: coarse grained model for biomolecular simulations. J. Phys. Chem. B 111, 7812–7824. 10.1021/jp071097f 17569554

[B49] Michaud-Agrawal N. Denning E. J. Woolf T. B. Beckstein O. (2011). Mdanalysis: a toolkit for the analysis of molecular dynamics simulations. J. Comput. Chem. 32, 2319–2327. 10.1002/jcc.21787 21500218 PMC3144279

[B50] Nosé S. (1984). A unified formulation of the constant temperature molecular dynamics methods. J. Chem. Phys. 81, 511–519. 10.1063/1.447334

[B51] Parrinello M. Rahman A. (1982). Strain fluctuations and elastic constants. J. Chem. Phys. 76, 2662–2666. 10.1063/1.443248

[B52] Periole X. Cavalli M. Marrink S.-J. Ceruso M. A. (2009). Combining an elastic network with a coarse-grained molecular force field: structure, dynamics, and intermolecular recognition. J. Chem. Theory Comput. 5, 2531–2543. 10.1021/ct9002114 26616630

[B53] Peulen T.-O. Hengstenberg C. S. Biehl R. Dimura M. Lorenz C. Valeri A. (2023). Integrative dynamic structural biology unveils conformers essential for the oligomerization of a large gtpase. eLife 12, e79565. 10.7554/elife.79565 37314846 PMC10374282

[B54] Prakash B. Renault L. Praefcke G. J. Herrmann C. Wittinghofer A. (2000). Triphosphate structure of guanylate-binding protein 1 and implications for nucleotide binding and gtpase mechanism. EMBO J. 19, 4555–4564. 10.1093/emboj/19.17.4555 10970849 PMC302049

[B55] Prakash B. Praefcke G. J. K. Renault L. Wittinghofer A. Herrmann C. (2000). Structure of human guanylate-binding protein 1 representing a unique class of gtp-binding proteins. Nature 403, 567–571. 10.1038/35000617 10676968

[B56] Qin Z. Fabre A. Buehler M. J. (2013). Structure and mechanism of maximum stability of isolated alpha-helical protein domains at a critical length scale. Eur. Phys. J. E 36, 53. 10.1140/epje/i2013-13053-8 23708839

[B57] Rivera-Cuevas Y. Clough B. Frickel E.-M. (2023). Human guanylate-binding proteins in intracellular pathogen detection, destruction, and host cell death induction. Curr. Opin. Immun. 84, 102373. 10.1016/j.coi.2023.102373 37536111

[B58] Rupper A. C. Cardelli J. A. (2008). Induction of guanylate binding protein 5 by gamma interferon increases susceptibility tosalmonella entericaserovar typhimurium-induced pyroptosis in raw 264.7 cells. Infect. Immun. 76, 2304–2315. 10.1128/iai.01437-07 18362138 PMC2423062

[B59] Santos J. C. Boucher D. Schneider L. K. Demarco B. Dilucca M. Shkarina K. (2020). Human gbp1 binds lps to initiate assembly of a caspase-4 activating platform on cytosolic bacteria. Nat. Commun. 11, 3276. 10.1038/s41467-020-16889-z 32581219 PMC7314798

[B60] Schrödinger L. L. C. (2025). The PyMOL molecular graphics system, version 3.0.

[B61] Schumann W. Strodel B. (2023). Allosteric communication induced by GTP binding sets off a closed-to-open transition in a bacterial dynamin-like protein. bioRxiv. 10.1101/2023.01.16.524228

[B62] Schumann W. Loschwitz J. Reiners J. Degrandi D. Legewie L. Stühler K. (2023). Integrative modeling of guanylate binding protein dimers. Protein Sci. 32, e4818. 10.1002/pro.4818 37916607 PMC10683561

[B63] Schwemmle M. Staeheli P. (1994). The interferon-induced 67-kda guanylate-binding protein (hgbp1) is a gtpase that converts gtp to gmp. J. Biol. Chem. 269, 11299–11305. 10.1016/s0021-9258(19)78125-3 7512561

[B64] Shenoy A. R. Wellington D. A. Kumar P. Kassa H. Booth C. J. Cresswell P. (2012). Gbp5 promotes nlrp3 inflammasome assembly and immunity in mammals. Science 336, 481–485. 10.1126/science.1217141 22461501

[B65] Shydlovskyi S. Zienert A. Y. Ince S. Dovengerds C. Hohendahl A. Dargazanli J. M. (2017). Nucleotide-dependent farnesyl switch orchestrates polymerization and membrane binding of human guanylate-binding protein 1. Proc. Natl. Acad. Sci. U. S. A. 114, E5559-E5568. 10.1073/pnas.1620959114 28645896 PMC5514708

[B66] Sistemich L. Stanchev L. D. Kutsch M. Roux A. Pomorski T. G. Herrmann C. (2021). Structural requirements for membrane binding of human guanylate-binding protein 1. FEBS J. 288, 4098–4114. 10.1111/febs.15703 33405388

[B67] Siuda I. Thøgersen L. (2013). Conformational flexibility of the leucine binding protein examined by protein domain coarse-grained molecular dynamics. J. Mol. Model. 19, 4931–4945. 10.1007/s00894-013-1991-9 24048570

[B68] Sousa da Silva A. W. Vranken W. F. (2012). Acpype - antechamber python parser interface. BMC Res. Notes 5, 367. 10.1186/1756-0500-5-367 22824207 PMC3461484

[B69] Syguda A. Bauer M. Benscheid U. Ostler N. Naschberger E. Ince S. (2012). Tetramerization of human guanylate-binding protein 1 is mediated by coiled-coil formation of the c-terminal alpha-helices. FEBS J. 279, 2544–2554. 10.1111/j.1742-4658.2012.08637.x 22607347

[B70] Tretina K. Park E.-S. Maminska A. MacMicking J. D. (2019). Interferon-induced guanylate-binding proteins: guardians of host defense in health and disease. J. Exp. Med. 216, 482–500. 10.1084/jem.20182031 30755454 PMC6400534

[B71] Truebestein L. Leonard T. A. (2016). Coiled-coils: the long and short of it. BioEssays 38, 903–916. 10.1002/bies.201600062 27492088 PMC5082667

[B72] Valdes-Tresanco M. S. Valdes-Tresanco M. E. Valiente P. A. Moreno E. (2021). gmx_MMPBSA: a new tool to perform end-state free energy calculations with GROMACS. J. Chem. Theory Comput. 17, 6281–6291. 10.1021/acs.jctc.1c00645 34586825

[B73] Vanommeslaeghe K. Hatcher E. Acharya C. Kundu S. Zhong S. Shim J. (2009). Charmm general force field: a force field for drug-like molecules compatible with the charmm all-atom additive biological force fields. J. Comput. Chem. 31, 671–690. 10.1002/jcc.21367 19575467 PMC2888302

[B74] Vöpel T. Syguda A. Britzen-Laurent N. Kunzelmann S. Lüdemann M.-B. Dovengerds C. (2010). Mechanism of gtpase-activity-induced self-assembly of human guanylate binding protein 1. J. Mol. Biol. 400, 63–70. 10.1016/j.jmb.2010.04.053 20450919

[B75] Wandel M. P. Pathe C. Werner E. I. Ellison C. J. Boyle K. B. von der Malsburg A. (2017). Gbps inhibit motility of Shigella flexneri but are targeted for degradation by the bacterial ubiquitin ligase ipah9.8. Cell Host Microbe 22, 507–518.e5. 10.1016/j.chom.2017.09.007 29024643 PMC5644667

[B76] Wang J. Wolf R. M. Caldwell J. W. Kollman P. A. Case D. A. (2004). Development and testing of a general amber force field. J. Comput. Chem. 25, 1157–1174. 10.1002/jcc.20035 15116359

[B77] Wang J. Wang W. Kollman P. A. Case D. A. (2006). Automatic atom type and bond type perception in molecular mechanical calculations. J. Mol. Graph. Model. 25, 247–260. 10.1016/j.jmgm.2005.12.005 16458552

[B78] Wassenaar T. A. Pluhackova K. Böckmann R. A. Marrink S. J. Tieleman D. P. (2014). Going backward: a flexible geometric approach to reverse transformation from coarse grained to atomistic models. J. Chem. Theory Comput. 10, 676–690. 10.1021/ct400617g 26580045

[B79] Wassenaar T. A. Pluhackova K. Moussatova A. Sengupta D. Marrink S. J. Tieleman D. P. (2015). High-throughput simulations of dimer and trimer assembly of membrane proteins. The daft approach. J. Chem. Theory Comput. 11, 2278–2291. 10.1021/ct5010092 26574426

[B80] Weismehl M. Chu X. Kutsch M. Lauterjung P. Herrmann C. Kudryashev M. (2024). Structural insights into the activation mechanism of antimicrobial gbp1. EMBO J. 43, 615–636. 10.1038/s44318-023-00023-y 38267655 PMC10897159

[B81] Xavier A. Al-Zeer M. A. Meyer T. F. Daumke O. (2020). hgbp1 coordinates chlamydia restriction and inflammasome activation through sequential gtp hydrolysis. Cell Rep. 31, 107667. 10.1016/j.celrep.2020.107667 32433976

[B82] Yu Y. Pan J. Liu M. Jiang H. Xiong J. Tao L. (2022). Guanylate-binding protein 2b regulates the ampk/mtor/ulk1 signalling pathway to induce autophagy during Mycobacterium bovis infection. Virulence 13, 875–889. 10.1080/21505594.2022.2073024 35531887 PMC9132469

[B83] Zhu S. Bradfield C. J. Maminska A. Park E.-S. Kim B.-H. Kumar P. (2024). Native architecture of a human gbp1 defense complex for cell-autonomous immunity to infection. Science 383, eabm9903. 10.1126/science.abm9903 38422126 PMC12091997

