# Supplementary material for: Mechanisms driving open-closed transitions and dimer stability of the large GTPases hGBP1 and hGBP5
Source: Front Mol Biosci. 2026 May 25;13:1732916. doi: 10.3389/fmolb.2026.1732916 (PMC13244007; doi:10.3389/fmolb.2026.1732916)
Supplement: Supplementary file 1 [file DataSheet1.pdf]

Supplementary Material

SUPPLEMENTARY FIGURES

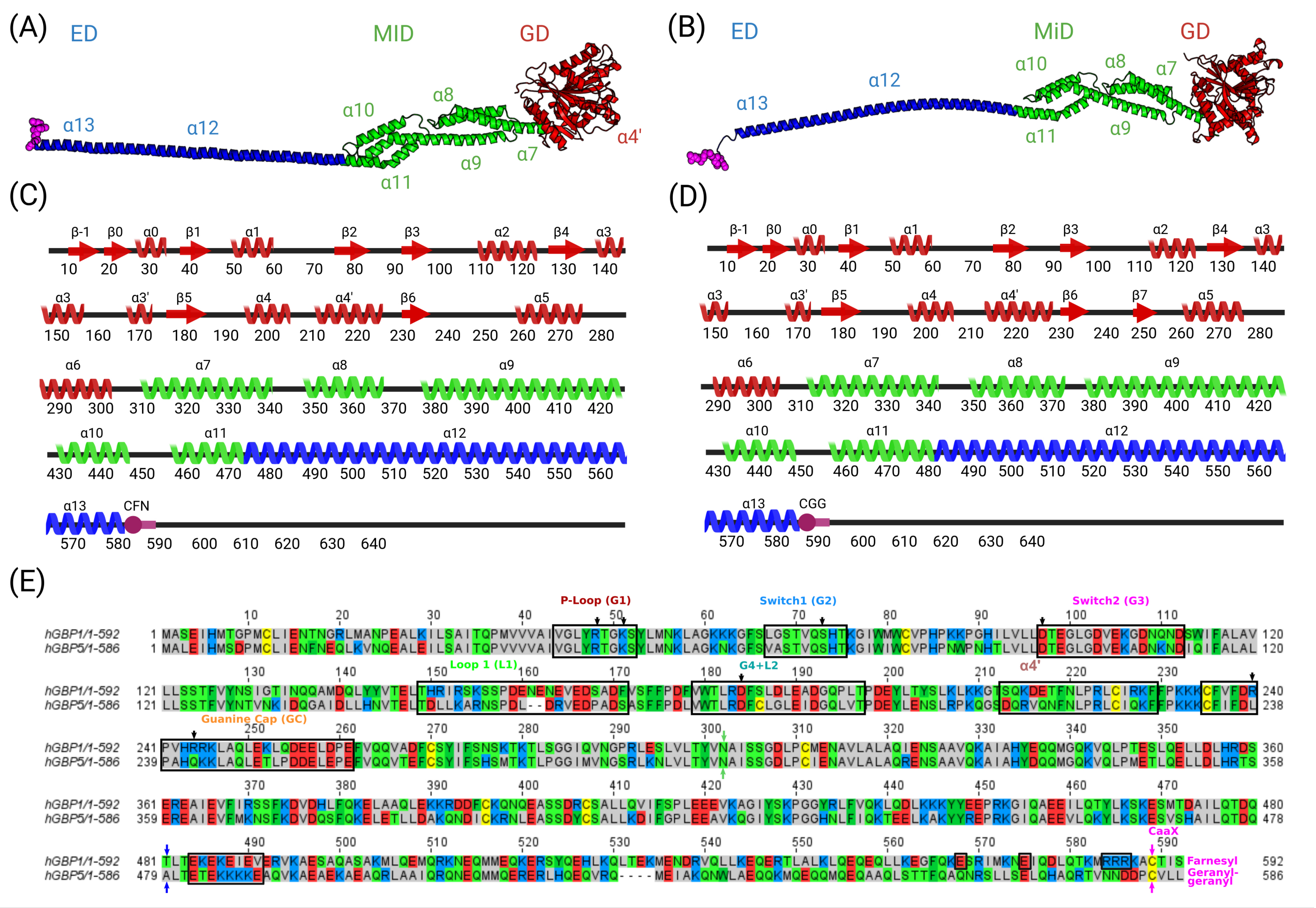

Figure S1. Secondary and primary structure of hGBP1 and hGBP5. The open conformation of **(A)** hGBP1 and **(B)** hGBP5 is shown as a cartoon, with the GD in red, MiD in green, ED in blue, and the respective prenyl group in magenta. The helices are labeled. Corresponding secondary structure diagrams are shown in **(C)** for hGBP1 (CFN for the farnesyl group) and **(D)** for hGBP5 (CGG for the geranylgeranyl group), along with residue numbers. In **(E)**, the sequences of hGBP1 (top) and hGBP5 (bottom) are provided, using green for polar, red for negatively charged, blue for positively charged, gray for neutral residues, and yellow for cysteine. Important regions are marked with boxes and labeled.

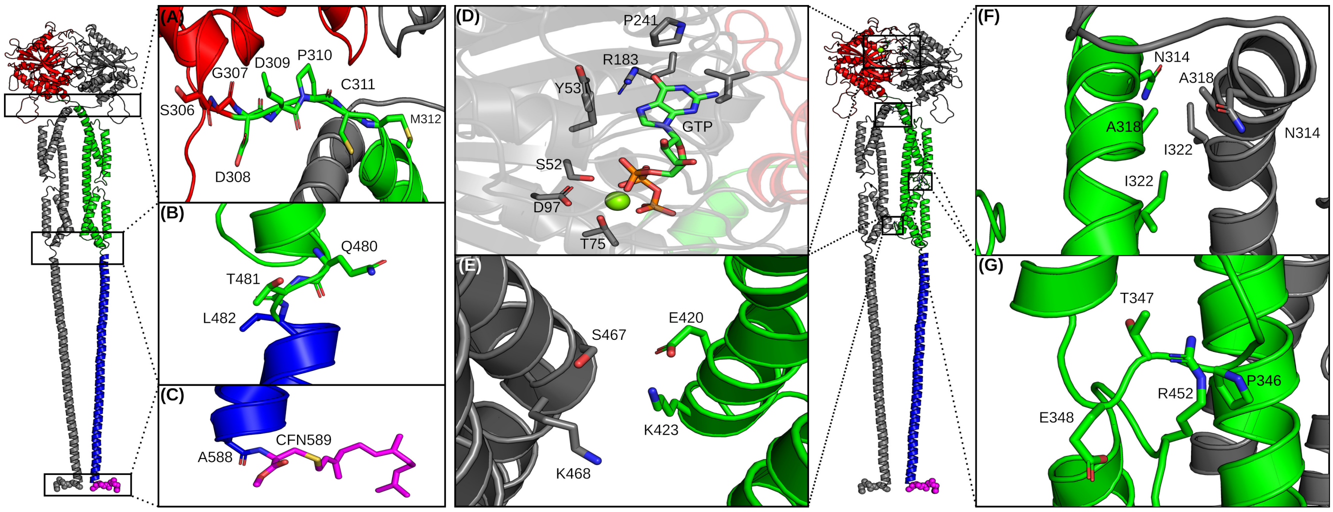

Figure S2. Challenges in building the open dimer model of hGBP1. The connections between the GD and MiD (**A**) and between the MiD and ED (**B**) were manually modeled using PyMol, as the alignment with the hGBP5 dimer template was not perfect. Care was taken to ensure that amino acids in opposing helices and loops did not clash; problematic regions are shown in (**E–G**). Additionally, the farnesyl group was attached manually (**C**), and GTP and Mg^{2+} were added to the GD, resulting in the holo-hGBP1 dimer.

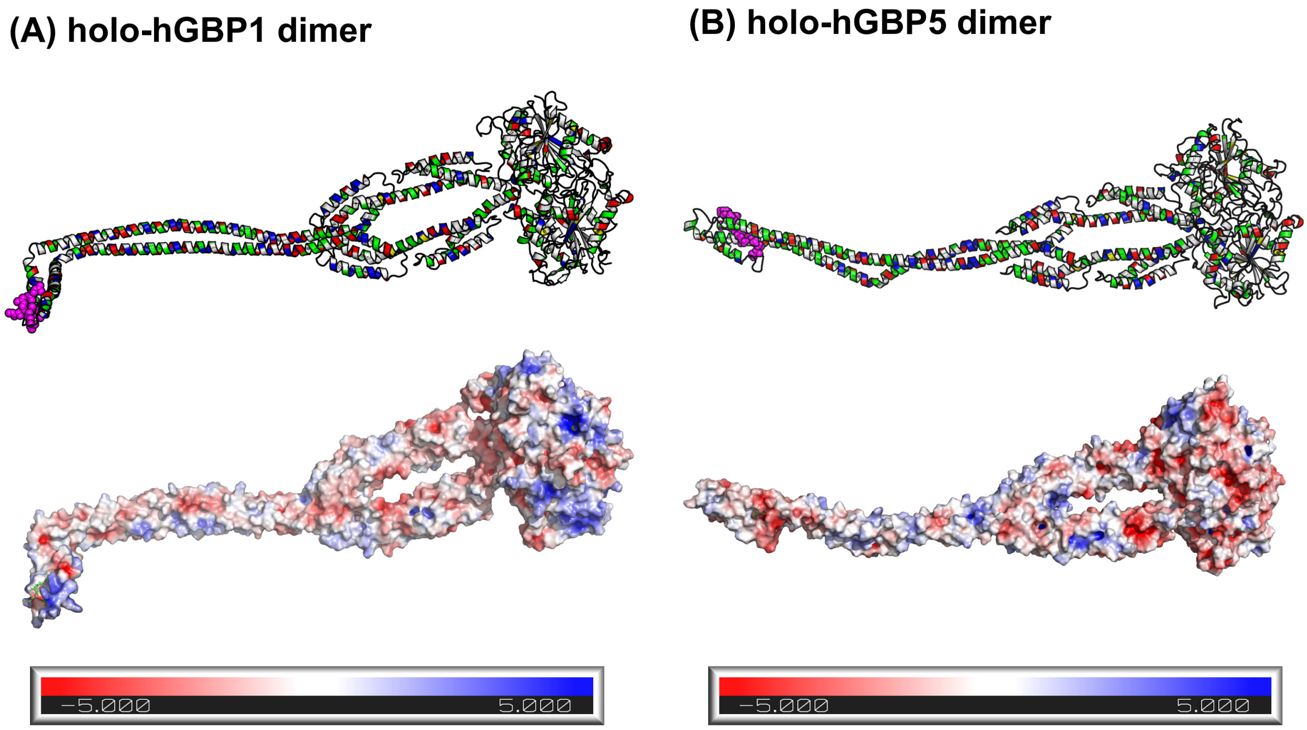

Figure S3. Charge distribution across the open hGBP1/5 dimers. Top: The proteins are shown as cartoons, with residues colored by amino acid type: green for polar, red for negatively charged, blue for positively charged, gray for neutral residues, and yellow for cysteine. **Bottom:** The electrostatic potential is mapped onto the protein surface, ranging from -5 (red) to $+5$ kT/e (blue).

(A) β-sheet restraints within the GD of the hGBP1 dimer **(B) β-sheet restraints within the GD of the hGBP5 dimer**

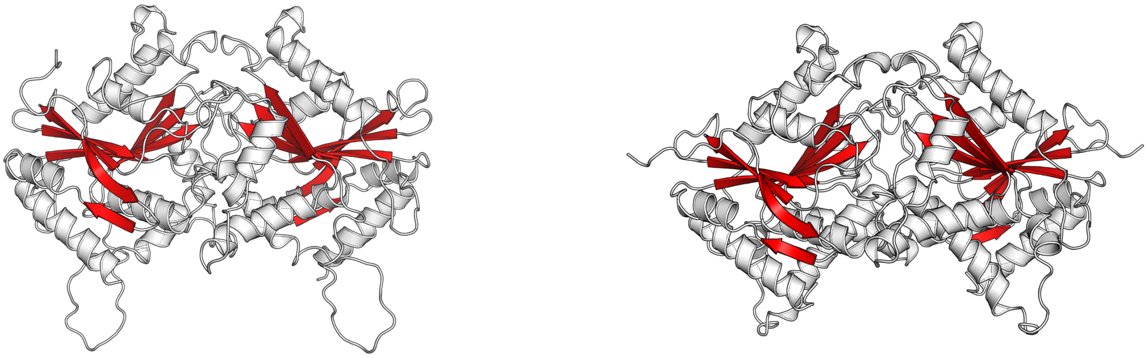

Figure S4. Visualization of G-domain backbone restraints The backbone atoms restrained during the AA-MD simulations are highlighted in red for both the **(A)** apo-hGBP1 and **(B)** apo-hGBP5 dimers. These restraints were applied on the β-sheets to avoid overall rotation of the hGBP proteins. The restrained residue ranges are: hGBP1: 11–17, 38–44, 77–84, 92–99, 125–131, 177–183, 232–238; hGBP5: 11–17, 38–45, 78–84, 92–98, 125–132, 175–181, 230–236. All restraints were applied with a force constant of 1000 kJ mol^{-1} nm^{-2}. All other residues in the GD were not restraint and are shown in white.

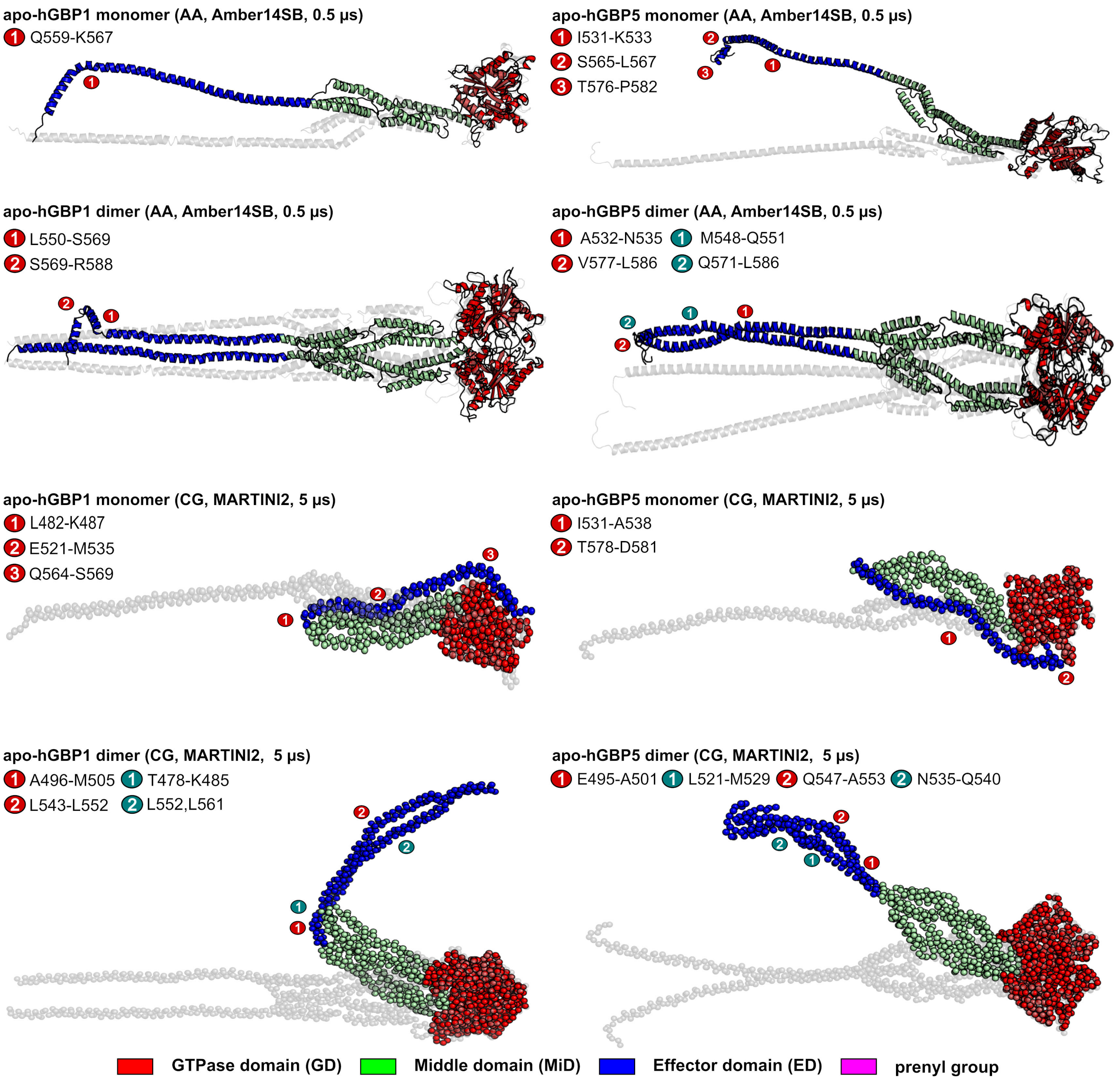

Figure S5. Start and end structures of the MD simulations of apo-hGBP1 (left) and apo-hGBP5 (right) monomers and dimers. Results are shown for the AA-MD (top two plots) and CG-MD (bottom two plots) simulations. The start structures are depicted in gray, while the end structures are shown in color. Regions where helices developed kinks, loops, or turns during the simulations are indicated by circles (red for the first protomer, cyan for the second protomer in the dimers), and the corresponding residue numbers are provided.

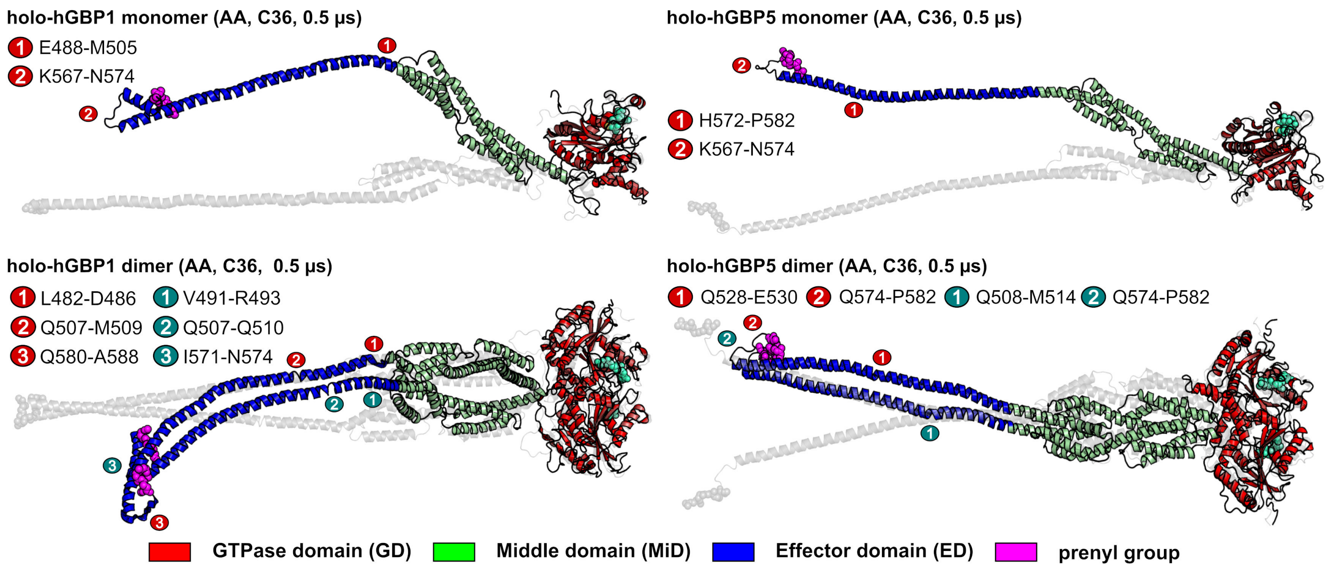

Figure S6. Start and end structures of the MD simulations of holo-hGBP1 (left) and holo-hGBP5 (right) monomers and dimers. Results are shown for AA-MD simulations with the CHARMM36 force field. The start structures are depicted in gray, while the end structures are shown in color. Regions where helices developed kinks, loops, or turns during the simulations are indicated by circles (red for the first protomer, cyan for the second protomer in the dimers), and the corresponding residue numbers are provided.

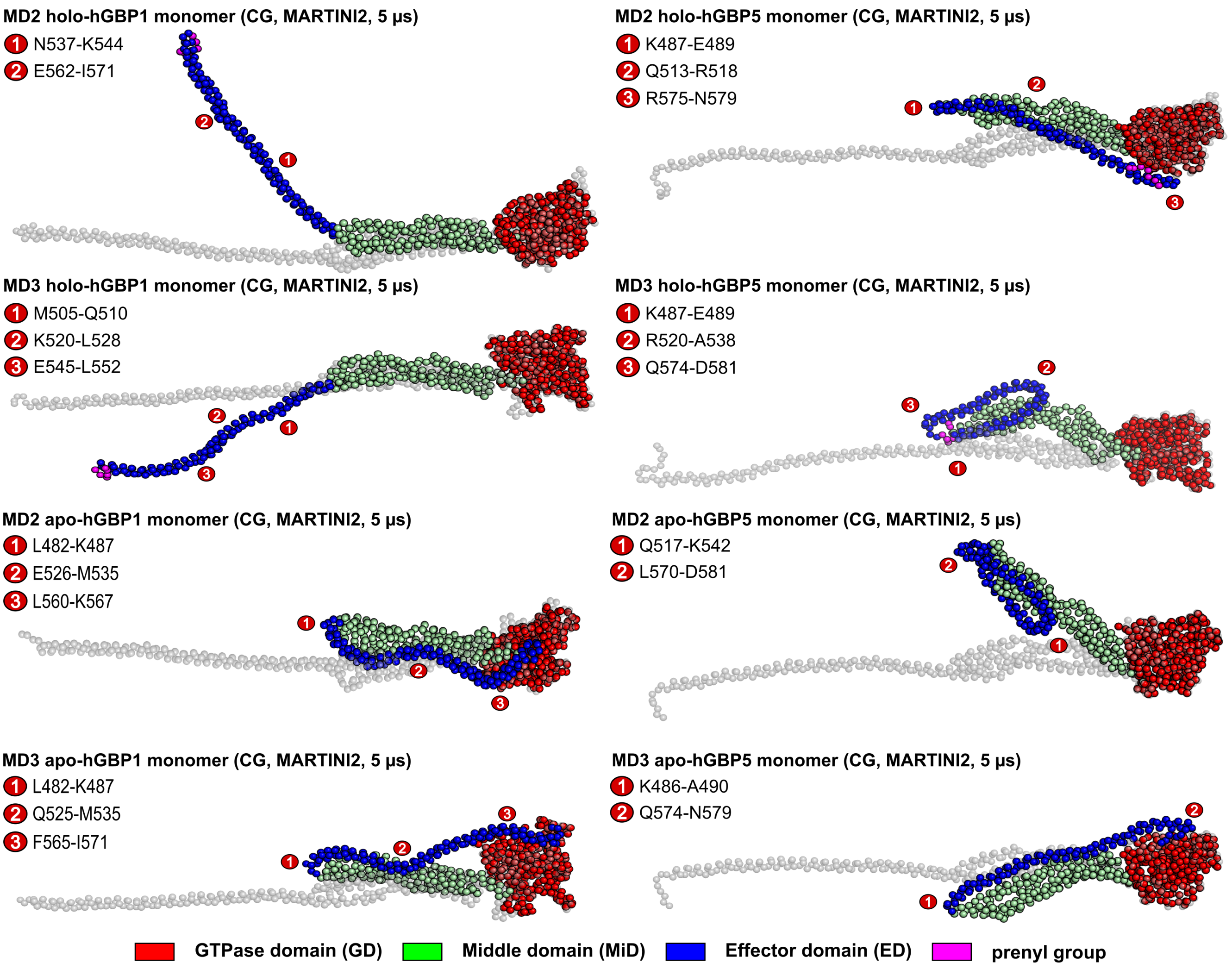

Figure S7. Start and end structures of the replicate CG-MD simulations of holo- and apo-hGBP1/5 monomers. Results are shown for the second and third (MD2 and MD3) CG-MD simulations. Results for the first CG-MD run are shown in Fig. 2 for the holo systems and in Fig. S5 for the apo systems. The start structures are depicted in gray, while the end structures are shown in color. Regions where helices developed kinks, loops, or turns during the simulations are indicated by red circles for the protein, and the corresponding residue numbers are provided.

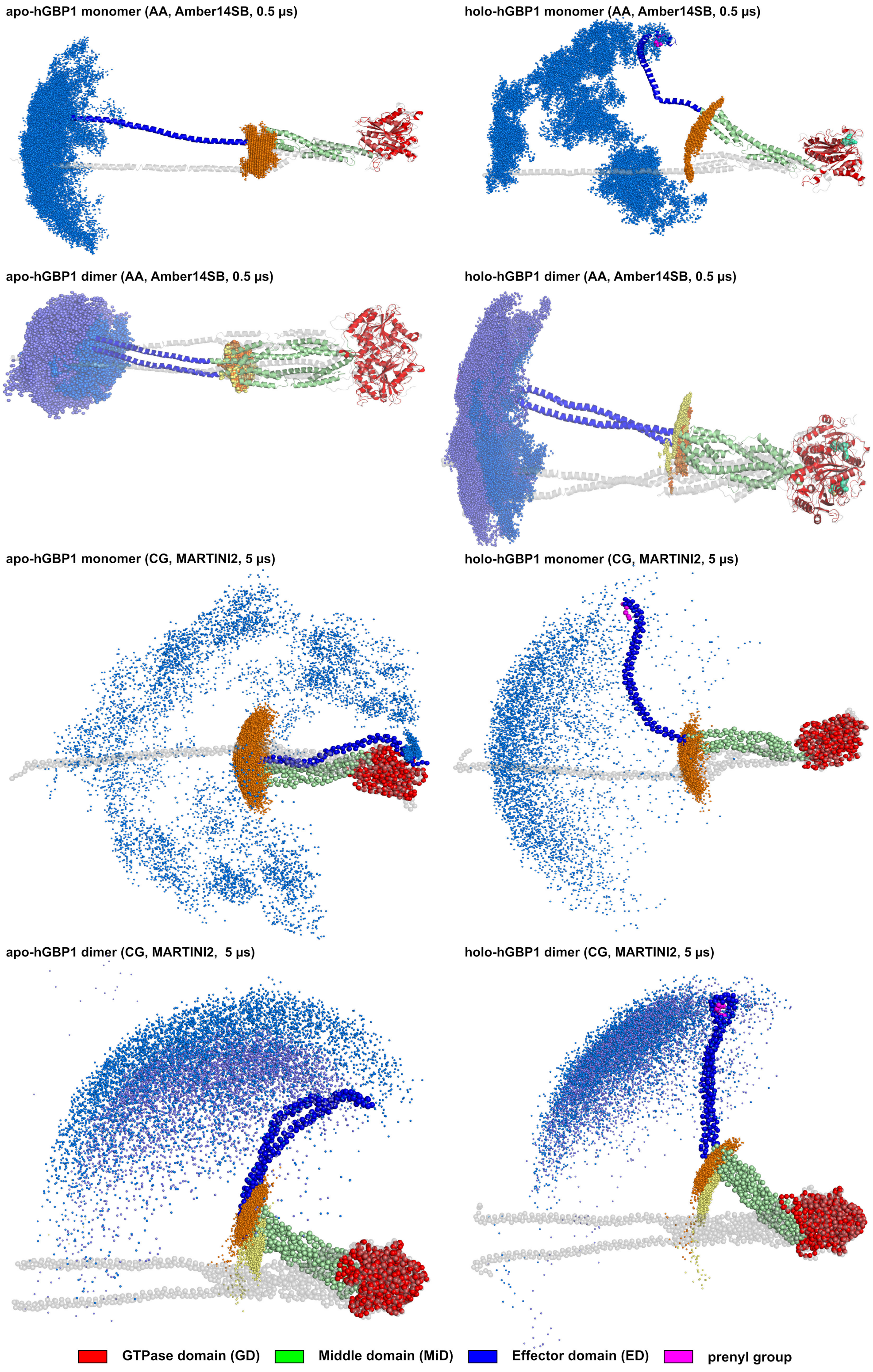

Figure S8. Spatial distribution of selected residues during the MD simulations of hGBP1. The motions of the MiD and ED are represented by the spatial distribution of residues Q480 (shown as orange dots for protomer 1 and yellow dots for protomer 2 in the dimer) for the MiD, and K582 (shown as blue dots for protomer 1 and purple dots for protomer 2 in the dimer) for the ED. Results are shown for all hGBP1 systems: monomer and dimer in apo and holo forms, simulated with AA-MD and CG-MD, respectively. The start structures are depicted as gray cartoons, and the final structures of the simulations as colored cartoons.

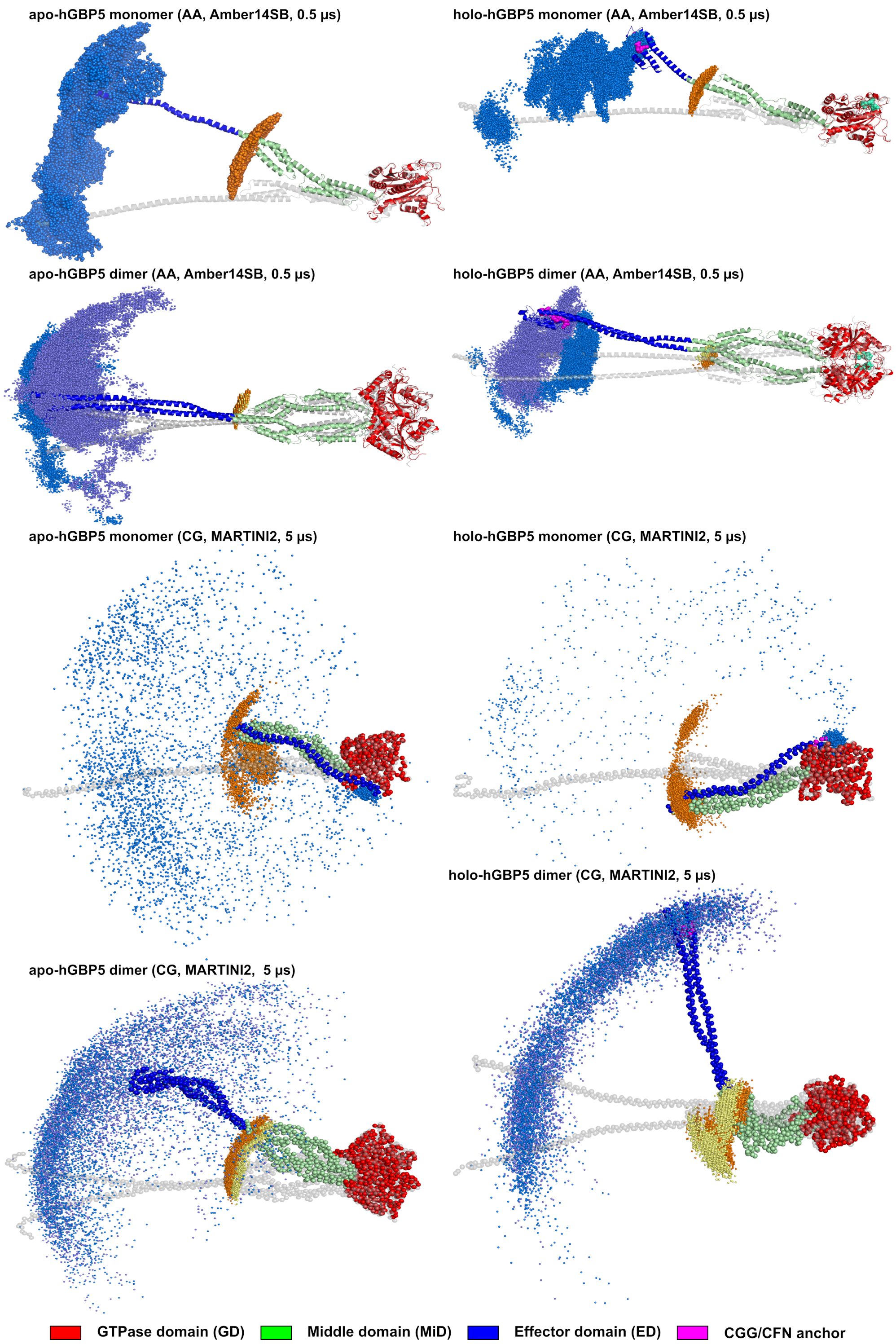

Figure S9. Spatial distribution of selected residues during the MD simulations of hGBP5. The motions of the MiD and ED are represented by the spatial distribution of residues Q480 (shown as orange dots for protomer 1 and yellow dots for protomer 2 in the dimer) for the MiD, and K582 (shown as blue dots for protomer 1 and purple dots for protomer 2 in the dimer) for the ED. Results are shown for all hGBP5 systems: monomer and dimer in apo and holo forms, simulated with AA-MD and CG-MD, respectively. The start structures are depicted as gray cartoons, and the final structures of the simulations as colored cartoons.

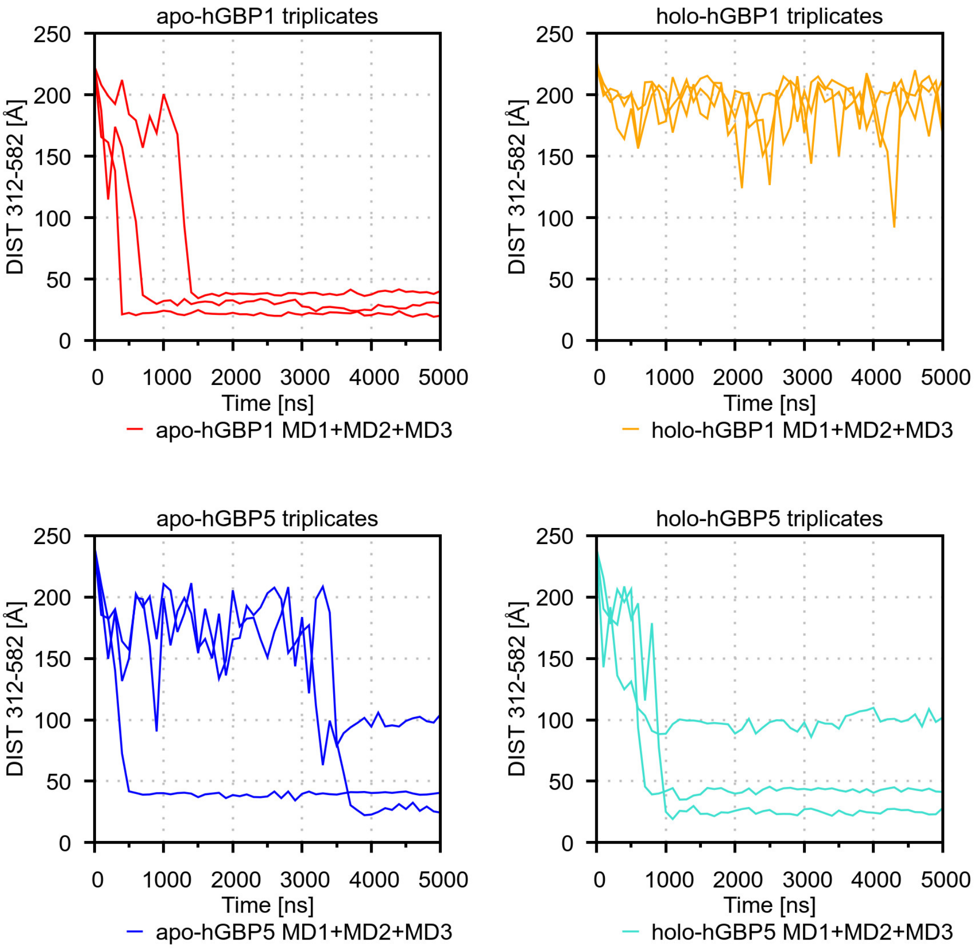

Figure S10. **Time evolution of the distance between residue 312 in the GD and residue 582 in the ED in apo-/holo-hGBP1 (red/orange) and apo-/holo-hGBP5 (blue/cyan) during the three CG-MD simulations per system.**

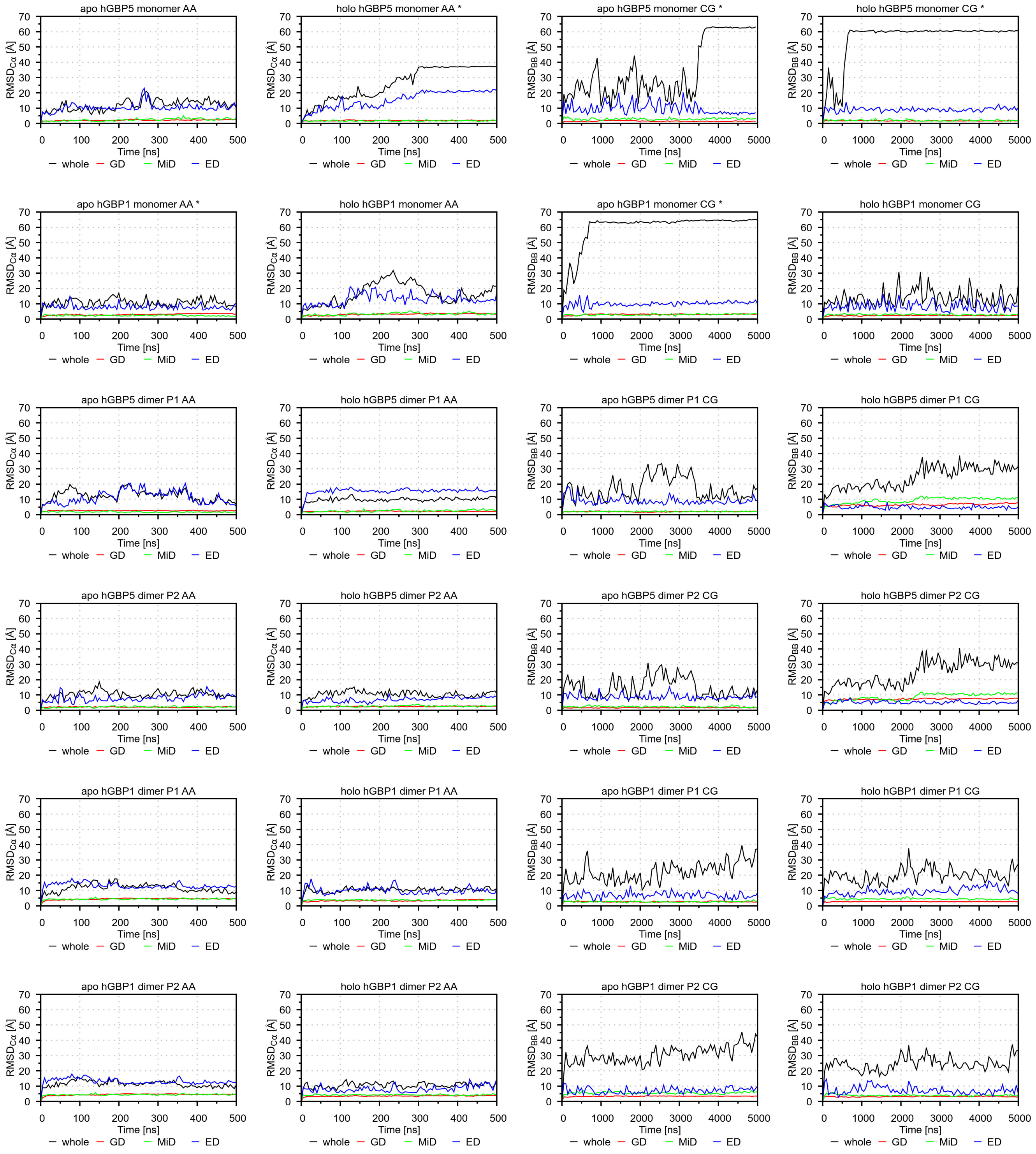

Figure S11. Time evolution of the RMSD in the simulations of hGBP1 and hGBP5. The RMSD was calculated with respect to the respective MD starting structure. The two left columns show results for AA-MD; the two right columns for the first of the three CG-MD runs per system. The first two rows represent monomer simulations, while the middle and last two rows correspond to protomer 1 (P1) and protomer 2 (P2) of the dimer simulations, respectively. Results are shown for both the apo and holo forms of the proteins. The RMSD for the entire protein (black) was calculated after alignment to the GD, while the RMSD of individual domains—GD (red), MiD (green), and ED (blue)—were computed after aligning each domain separately. Systems that reached a new stable state are marked with an asterisk above the plot.

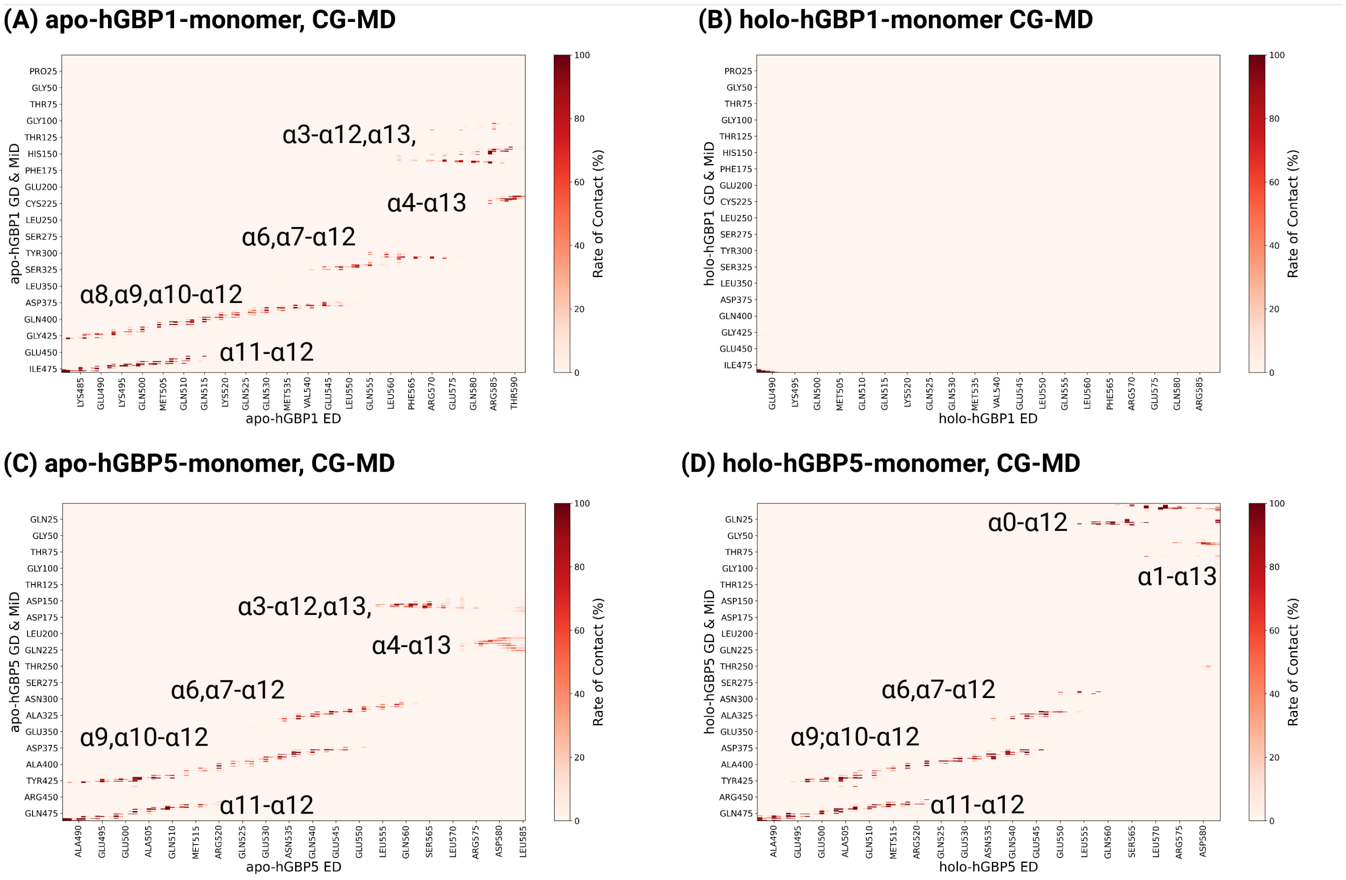

Figure S12. Rate of contacts between the ED and the GD/MiD in hGBP1 (top) and hGBP5 (bottom) monomers. Results are shown for the last 0.5 μs of the CG-MD simulations for **A/B** apo-/holo-hGBP1 and **C/D** apo-/holo-hGBP5. Relevant contact areas are labeled. For holo-hGBP1 no contacts are formed as no closing occurred during the simulation.

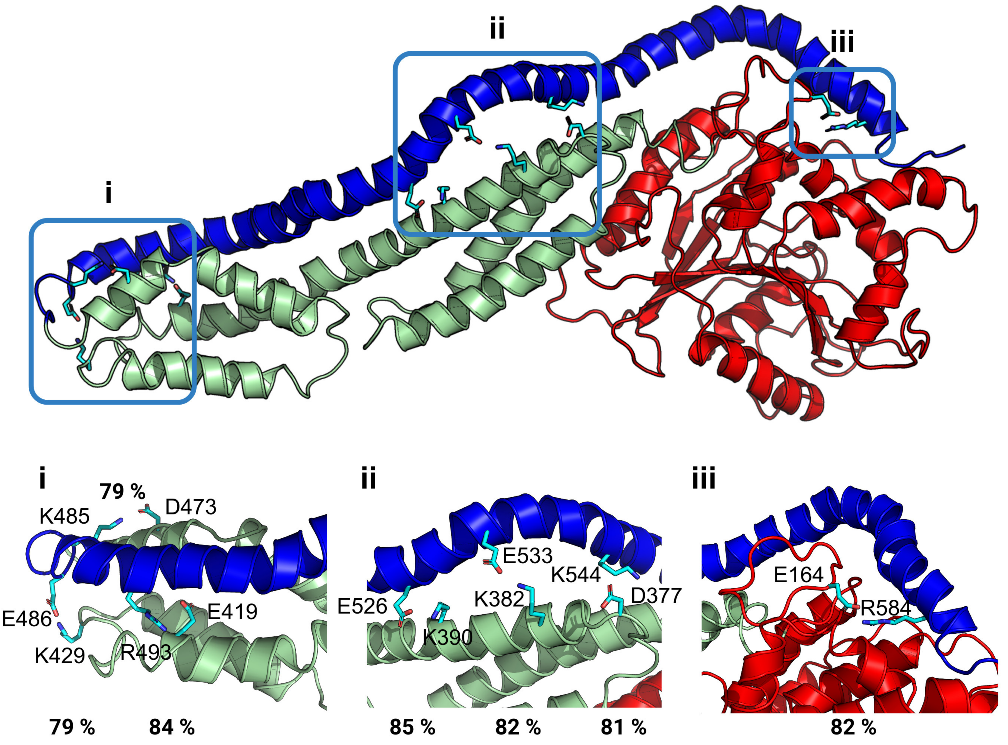

Figure S13. Key interactions driving the open-to-closed transition in the apo-hGBP1 monomer during CG-MD. The protein is shown as a cartoon, with the GD in red, the MiD in green, and the ED in blue. The three regions (i) to (iii), which are of particular relevance, are shown as zoom-ins below the protein. Key residues are depicted as sticks with standard atom-type coloring and labeled by residue number; for each salt bridge, the interaction occupancy is provided as a percentage of the total trajectory duration. The structural representation shows the final frame of a representative CG-MD simulation, back-mapped to the all-atom level from one of three independent replicate trajectories.

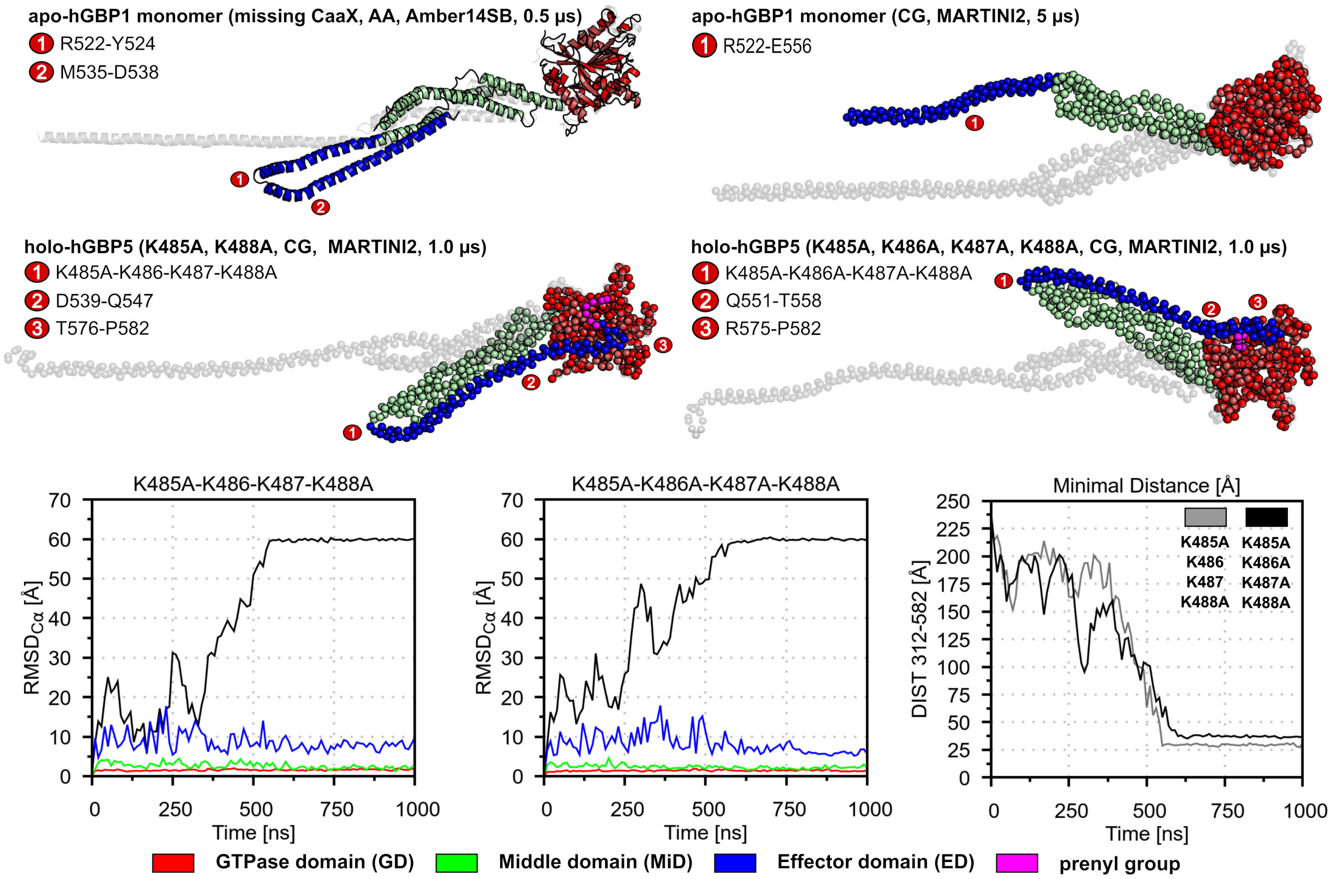

Figure S14. Mutational analysis of the conformational closing in apo-hGBP1 and holo-hGBP5 monomers. (Top) Initial and final structures from AA-MD (left) and CG-MD (right) simulations of apo-hGBP1-ΔCTIS (lacking residues 589–592). While a helical break is observed in the AA-MD, no open-to-closed transition occurred in either model. **(Middle)** Start and end structures of 1 μs CG-MD simulations for mutated holo-hGBP5 variants. **(Bottom)** Convergence and domain dynamics in holo-hGBP5 mutants. RMSD plots indicate that new stable states are reached, with the closing transition completing after approximately 0.6 μs in both mutational runs, as shown by the minimal distance between residues 312 and 582.

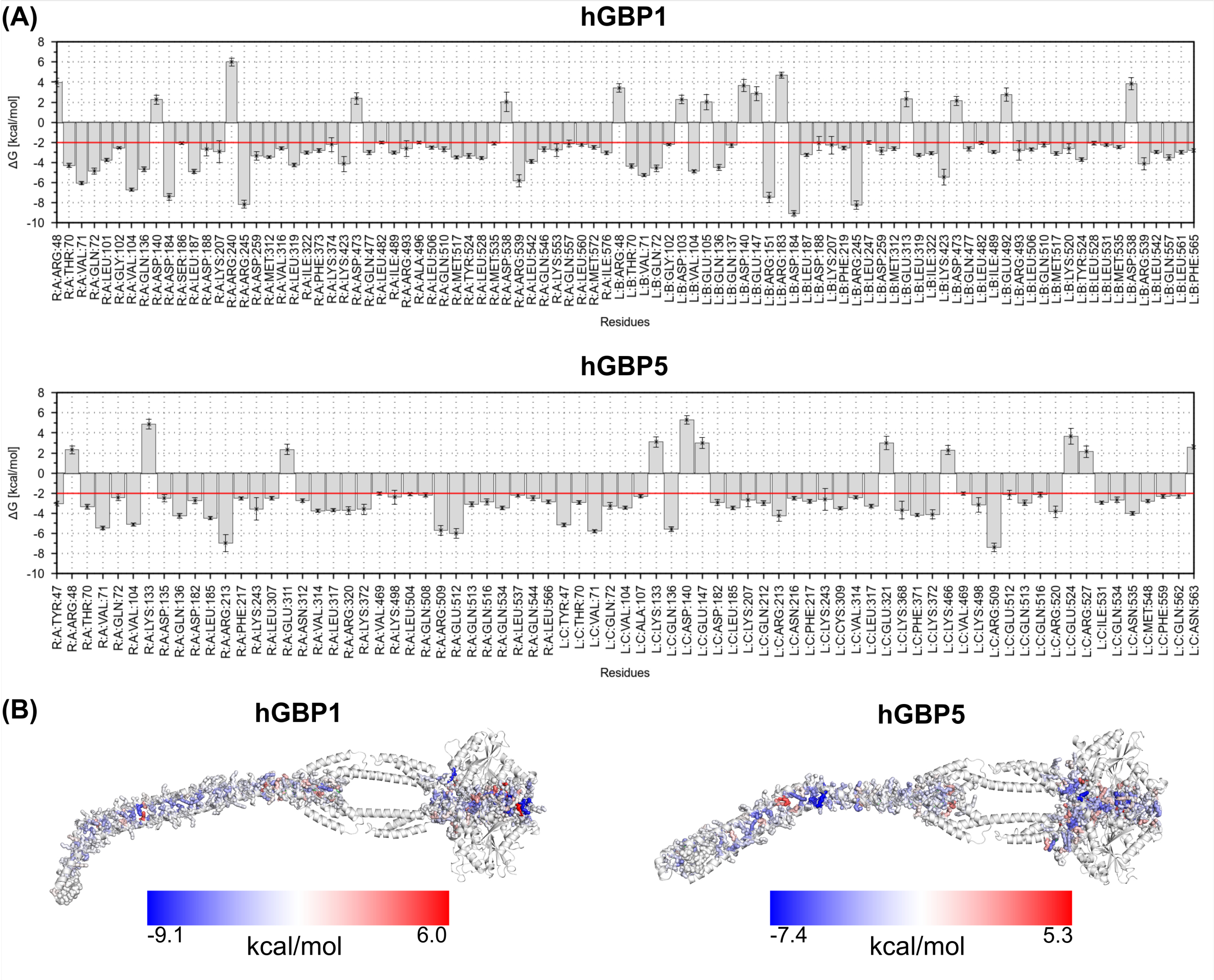

Figure S15. Residual contributions to the binding free energy ΔG between the two protomers of holo-hGBP1 and holo-hGBP5 dimers. The binding free energy was calculated using the gmxMMPBSA tool applied to the final 100 ns of the 500 ns AA-MD trajectories using 1,000 frames sampled at 100 ps intervals **A** Time-averaged free energies (with standard deviations as error bars) are shown for residues with ΔG values above or below ± 2 kcal/mol, displayed as bar plots. ”R:A:” and ”L:C:” indicate residues belonging to protomer P1 and P2, respectively. **B** The residual ΔG values are color-mapped onto the protein surface, with negative values up to zero shown in shades of blue, transitioning to white at zero, and increasing in red for positive values. The color spectrum’s minimum and maximum correspond to the lowest and highest residual ΔG values (in kcal/mol) observed in the respective trajectory.

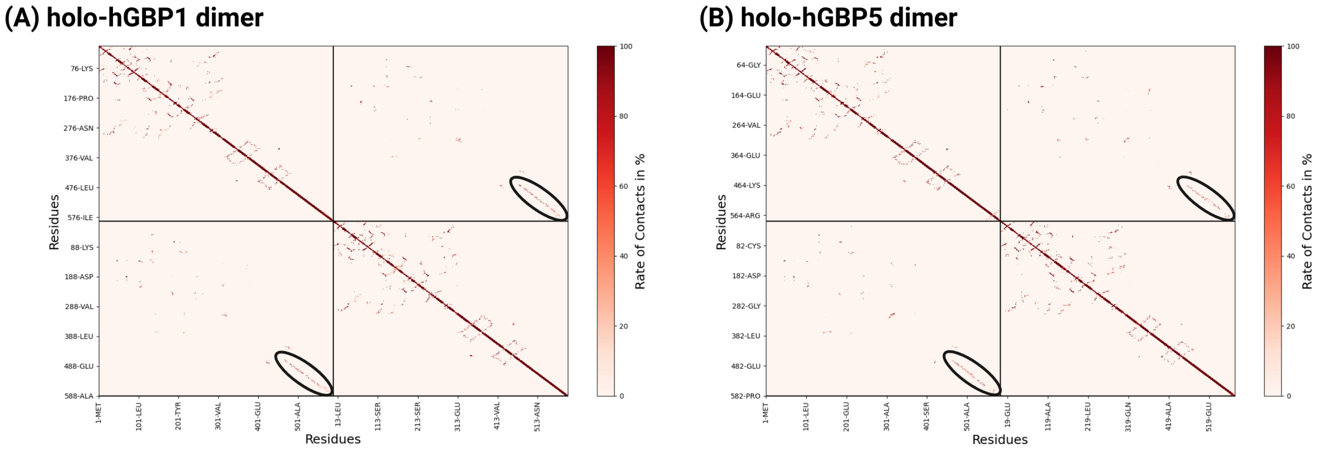

Figure S16. Rate of intra- and inter-protein contacts in hGBP1 and hGBP5 dimers. Results are shown for the full AA-MD trajectories (using the Amber14SB force field) of **A** the holo-hGBP1 dimer and **B** the holo-hGBP5 dimer. Intra-protein contacts are represented in the upper-left and lower-right quadrants, while the off-diagonal blocks illustrate the inter-protomer interactions. The specific contacts between the two effector domains (EDs) that facilitate coiled-coil formation are highlighted with ellipsoids.

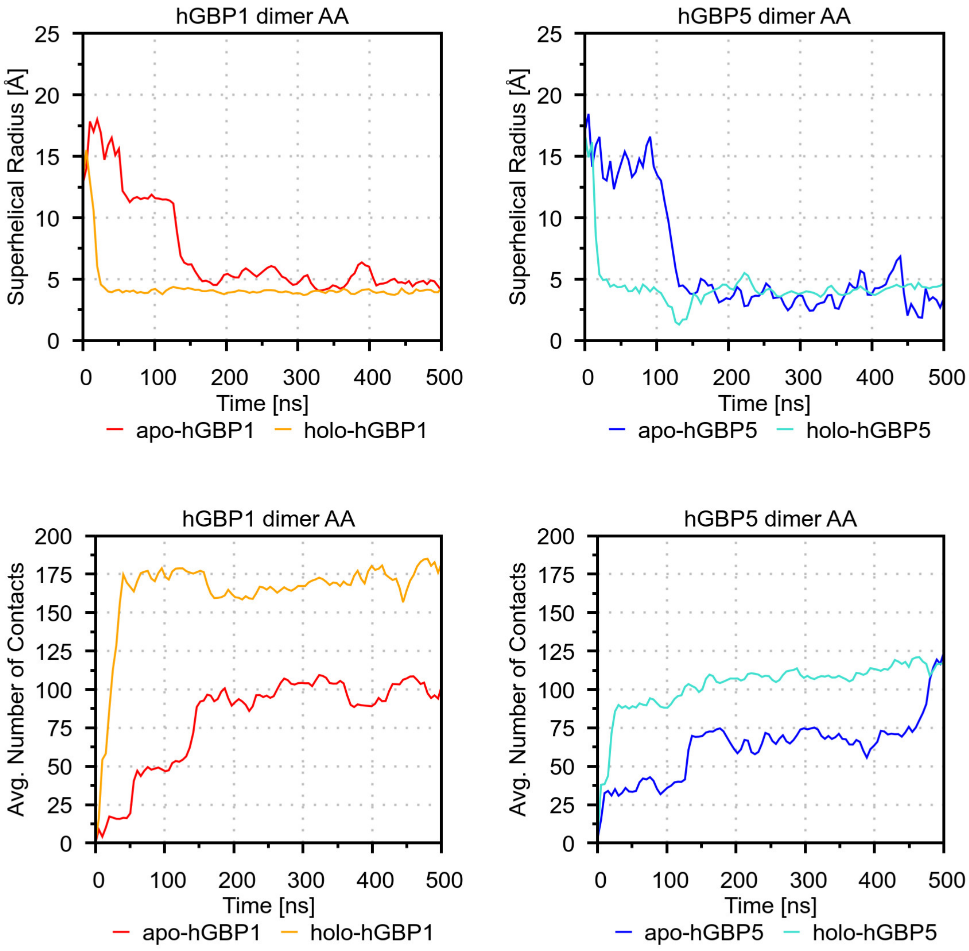

Figure S17. Quantitative analysis of coiled-coil formation in AA-MD simulations. Evolution of the superhelical radius, R_0 (top), and the number of inter-protomer contacts (bottom) between the two EDs during the all-atom MD trajectories of hGBP1 (left) and hGBP5 (right). Contact counts were determined using a 4 Å heavy-atom distance cutoff. A decrease in R_0 to $\sim$5 Å correlates with an increase in contact density, signaling the maturation of the coiled-coil interface.

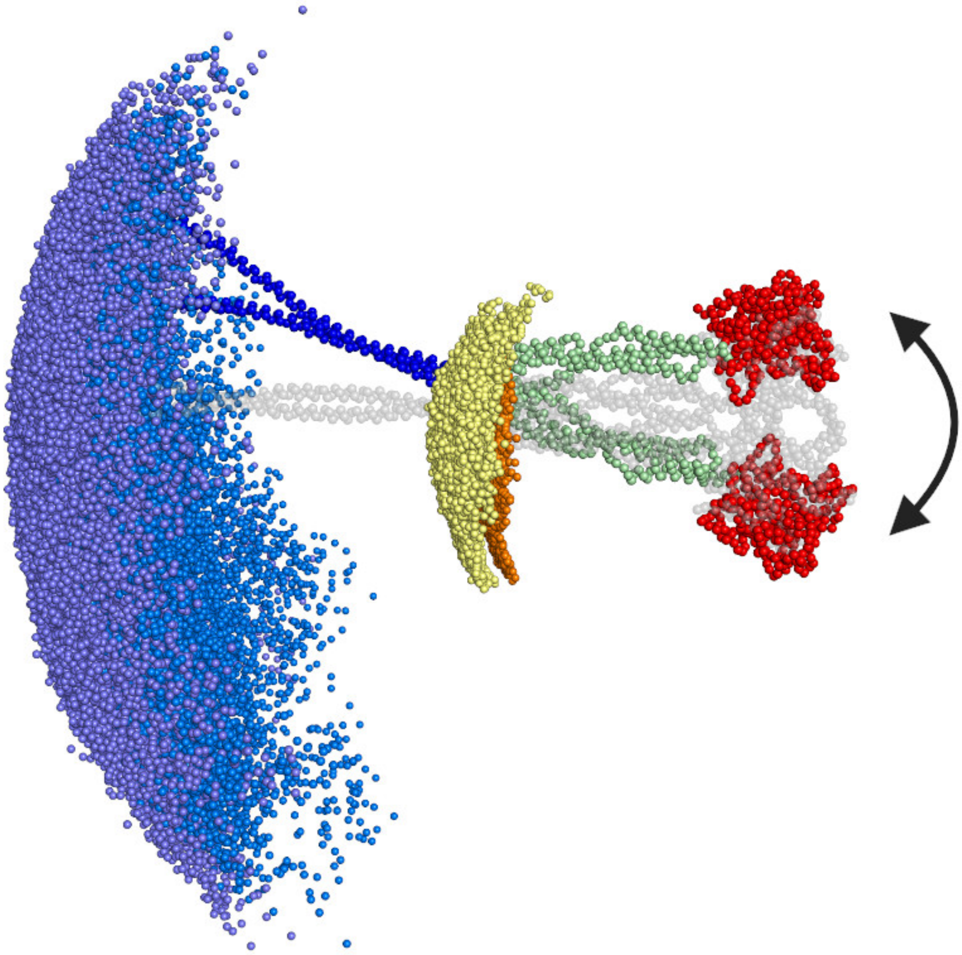

Figure S18. Instability of the open mGBP2 dimer without domain swapping. A 5 μs CG-MD simulation of the open mGBP2 dimer—where the MiD and ED are aligned parallel but do not cross (predicted by AlphaFold)—revealed instability. This is depicted by overlaying the initial (gray) and final (colored) structures, along with the spatial distribution of residues L480 (P1: orange dots; P2: yellow dots) at the MiD–ED transition, and S582 (P1: blue dots; P2: purple dots) at the ED tip. Most importantly, the GD–GD interface predicted by Alphafold—differing from that in hGBP1 and hGBP5 dimers—is unstable, causing the two GDs and also MiDs to move apart during the simulation.

SUPPLEMENTARY TABLES

Table S1. Salt-bridge contacts in apo-hGBP1, apo-hGBP5, and holo-hGBP5 monomers between the ED and GD/MiD as a result of open-to-closed transitions in CG-MD simulations. Only salt bridges with a probabilities of at least 30% for apo-hGBP1 and 10% for apo-hGBP5 and holo-hGBP5 are shown to focus on the most relevant ones.

apo-hGBP1 monomer	apo-hGBP5 monomer	holo-hGBP5 monomer
112:ASP - 585:ARG 30.60 %	159:ASP - 564:ARG 26.80 %	4:GLU - 572:HIS 82.16 %
147:GLU - 584:ARG 77.40 %	161:ASP - 564:ARG 14.46 %	9:ASP - 572:HIS 24.64 %
147:GLU - 585:ARG 71.64 %	320:ARG - 545:GLU 21.06 %	9:ASP - 575:ARG 47.54 %
159:ASP - 562:LYS 55.68 %	389:LYS - 530:GLU 24.62 %	26:GLU - 564:ARG 81.82 %
159:ASP - 573:LYS 60.14 %	392:ASP - 533:LYS 22.08 %	29:GLU - 564:ARG 81.68 %
160:GLU - 570:ARG 45.00 %	396:ARG - 530:GLU 21.26 %	57:LYS - 580:ASP 10.56 %
160:GLU - 573:LYS 51.12 %	399:GLU - 520:ARG 23.44 %	61:LYS - 580:ASP 69.68 %
162:GLU - 573:LYS 49.96 %	399:GLU - 527:ARG 16.76 %	61:LYS - 581:ASP 62.42 %
162:GLU - 584:ARG 76.48 %	403:ASP - 520:ARG 28.76 %	63:LYS - 580:ASP 54.04 %
162:GLU - 585:ARG 36.88 %	403:ASP - 527:ARG 11.56 %	63:LYS - 581:ASP 71.32 %
164:GLU - 584:ARG 81.88 %	410:LYS - 517:GLU 23.36 %	290:ARG - 550:GLU 65.12 %
164:GLU - 587:LYS 40.58 %	417:GLU - 509:ARG 17.26 %	320:ARG - 550:GLU 85.30 %
166:GLU - 584:ARG 36.08 %	427:LYS - 495:GLU 30.16 %	380:LYS - 539:GLU 57.00 %
217:GLU - 587:LYS 30.40 %	466:LYS - 512:GLU 18.46 %	381:GLU - 542:LYS 81.00 %
308:ASP - 570:ARG 35.60 %	467:GLU - 509:ARG 29.54 %	392:ASP - 533:LYS 76.88 %
377:ASP - 544:LYS 81.10 %	482:GLU - 487:LYS 48.34 %	395:LYS - 530:GLU 25.84 %
377:ASP - 548:ARG 68.54 %	482:GLU - 488:LYS 34.30 %	399:GLU - 518:ARG 43.20 %
382:LYS - 533:GLU 82.02 %	484:GLU - 487:LYS 89.04 %	399:GLU - 522:HIS 60.90 %
382:LYS - 536:GLU 43.88 %	484:GLU - 488:LYS 56.84 %	399:GLU - 527:ARG 11.34 %
383:GLU - 534:LYS 63.64 %	485:LYS - 489:GLU 10.54 %	403:ASP - 518:ARG 54.28 %
389:GLU - 529:LYS 50.44 %	486:LYS - 489:GLU 68.52 %	403:ASP - 522:HIS 25.38 %
390:LYS - 526:GLU 85.50 %		410:LYS - 512:GLU 46.60 %
390:LYS - 533:GLU 31.98 %		410:LYS - 519:GLU 12.00 %
393:ASP - 522:ARG 71.58 %		421:LYS - 512:GLU 52.90 %
394:ASP - 522:ARG 40.44 %		427:LYS - 497:GLU 24.04 %
397:LYS - 518:GLU 69.52 %		457:GLU - 522:HIS 24.10 %
401:GLU - 511:ARG 37.96 %		461:LYS - 517:GLU 51.42 %
401:GLU - 512:LYS 64.98 %		461:LYS - 519:GLU 27.62 %
405:ASP - 504:LYS 57.18 %		466:LYS - 512:GLU 44.58 %
405:ASP - 512:LYS 57.52 %		471:HIS - 500:GLU 27.50 %
419:GLU - 493:ARG 83.58 %		482:GLU - 487:LYS 92.90 %
420:GLU - 493:ARG 71.94 %		482:GLU - 488:LYS 13.98 %
421:GLU - 493:ARG 31.38 %		482:GLU - 493:LYS 70.40 %
423:LYS - 486:GLU 65.64 %		484:GLU - 487:LYS 44.04 %
423:LYS - 490:GLU 71.66 %		486:LYS - 489:GLU 55.48 %
429:LYS - 486:GLU 78.90 %		
459:GLU - 511:ARG 55.20 %		
468:LYS - 497:GLU 70.12 %		
473:ASP - 485:LYS 76.24 %		

Table S2. Coiled-coil parameters and salt-bridge contacts in apo -and holo-hGBP1/5 dimers. Coiled-coil parameters were calculated for the last AA-MD snapshot using the online tool `http://www.gevorggrigoryan.com/cccp/`. Salt-bridge probabilities between the two EDs were calculated for the AA-MD trajectories using a distance cutoff of 4 Å to identify salt bridges. Only salt bridges with $\geq$ 5% probability are listed.

apo-hGBP1 Dimer	holo-hGBP1 Dimer	apo-hGBP5 Dimer	holo-hGBP5 Dimer
R_0 = 3.65 Å<br>Pitch = 111.6 Å<br>Pitch angle α = 11.6°	R_0 = 5.67 Å<br>Pitch = 122.6 Å<br>Pitch angle α = 6.3°	R_0 = 3.47 Å<br>Pitch = 124.7 Å<br>Pitch angle α = 9.9°	R_0 = 4.70 Å<br>Pitch = 129.4 Å<br>Pitch angle α = 5.1°
A:484-GLU - B:485-LYS 56.9%	A:485-LYS - B:486-GLU 75.2%	A:493-LYS - B:495-GLU 7.3%	A:487-LYS - B:489-GLU 9.7%
A:485-LYS - B:484-GLU 28.3%	A:486-GLU - B:485-LYS 70.4%	A:497-GLU - B:498-LYS 99.0%	A:489-GLU - B:487-LYS 9.7%
A:488-GLU - B:485-LYS 71.0%	A:488-GLU - B:493-ARG 30.1%	A:498-LYS - B:497-GLU 91.0%	A:497-GLU - B:498-LYS 97.6%
A:490-GLU - B:485-LYS 7.1%	A:492-GLU - B:493-ARG 97.4%	A:500-GLU - B:498-LYS 8.8%	A:498-LYS - B:497-GLU 89.5%
A:495-LYS - B:488-GLU 86.8%	A:493-ARG - B:488-GLU 18.8%	A:509-ARG - B:512-GLU 98.7%	A:498-LYS - B:500-GLU 12.2%
A:495-LYS - B:492-GLU 59.9%	A:493-ARG - B:492-GLU 97.9%	A:512-GLU - B:509-ARG 97.8%	A:500-GLU - B:498-LYS 17.8%
A:520-LYS - B:514-GLU 66.5%	A:497-GLU - B:495-LYS 7.0%	A:519-GLU - B:520-ARG 39.5%	A:509-ARG - B:512-GLU 99.7%
A:520-LYS - B:518-GLU 23.6%	A:518-GLU - B:520-LYS 5.2%	A:519-GLU - B:527-ARG 5.2%	A:512-GLU - B:509-ARG 98.0%
A:520-LYS - B:521-GLU 7.9%	A:520-LYS - B:521-GLU 44.9%	A:520-ARG - B:519-GLU 36.4%	A:512-GLU - B:520-ARG 53.8%
A:521-GLU - B:520-LYS 24.6%	A:521-GLU - B:520-LYS 92.5%	A:527-ARG - B:530-GLU 62.4%	A:519-GLU - B:520-ARG 39.2%
A:527-HIS - B:521-GLU 64.9%	A:538-ASP - B:539-ARG 75.2%	A:530-GLU - B:527-ARG 29.4%	A:519-GLU - B:527-ARG 46.6%
A:539-ARG - B:538-ASP 7.9%	A:539-ARG - B:538-ASP 93.7%	A:542-LYS - B:545-GLU 49.7%	A:520-ARG - B:519-GLU 28.6%
A:545-GLU - B:539-ARG 57.7%	A:553-LYS - B:556-GLU 83.3%	A:545-GLU - B:542-LYS 63.2%	A:524-GLU - B:527-ARG 13.1%
A:553-LYS - B:545-GLU 60.5%	A:556-GLU - B:553-LYS 80.9%		A:527-ARG - B:530-GLU 95.8%
A:556-GLU - B:553-LYS 51.3%	A:567-LYS - B:558-GLU 18.3%		
A:563-GLU - B:553-LYS 20.6%	A:568-GLU - B:567-LYS 34.6%		
A:567-LYS - B:556-GLU 5.4%	A:570-ARG - B:558-GLU 4.5%		
A:568-GLU - B:582-LYS 96.7%	A:573-LYS - B:568-GLU 10.9%		
A:568-GLU - B:585-ARG 95.7%	A:587-LYS - B:575-GLU 7.9%		
A:568-GLU - B:586-ARG 97.6%			
A:575-GLU - B:582-LYS 19.3%			
A:575-GLU - B:586-ARG 95.2%			
